# An HLA-G/SPAG9/STAT3 axis promotes brain metastases

**DOI:** 10.1073/pnas.2205247120

**Published:** 2023-02-13

**Authors:** Blessing Iquo Bassey-Archibong, Chirayu Rajendra Chokshi, Nikoo Aghaei, Agata Monika Kieliszek, Nazanin Tatari, Dillon McKenna, Mohini Singh, Minomi Kalpana Subapanditha, Arun Parmar, Daniel Mobilio, Neil Savage, Fred Lam, Tomas Tokar, John Provias, Yu Lu, Shawn Christopher Chafe, Charles Swanton, Robert Edward Hynds, Chitra Venugopal, Sheila Kumari Singh

**Affiliations:** ^a^Department of Surgery, McMaster University, Hamilton, ON, L8S 4K1, Canada; ^b^Department of Biochemistry and Biomedical Sciences, McMaster University, Hamilton, ON, L8S 4K1, Canada; ^c^Department of Surgery, Division of Neurosurgery, McMaster University Faculty of Health Sciences, Hamilton General Hospital, Hamilton, ON, L8S 4K1, Canada; ^d^Osteoarthritis Research Program, Division of Orthopedic Surgery, Schroeder Arthritis Institute, University Health Network, Toronto, ON, M5T 2S8, Canada; ^e^Data Science Discovery Centre for Chronic Diseases, Krembil Research Institute, University Health Network, Toronto, ON, M5T 2S8, Canada; ^f^Department of Anatomical Pathology (Neuropathology), Hamilton General Hospital, Hamilton, ON, L8L 2X2, Canada; ^g^Department of Pathology and Molecular Medicine, Faculty of Health Sciences, McMaster University, Hamilton, ON, L8S 4K1, Canada; ^h^The Cancer Research UK (CRUK) Lung Cancer Centre of Excellence, University College London (UCL) Cancer Institute, University College London, London, WC1E 6DD, United Kingdom; ^i^Cancer Evolution and Genome Instability Laboratory, The Francis Crick Institute, London, NW1 1AT, United Kingdom

**Keywords:** HLA-G, SPAG9, STAT3, brain metastases

## Abstract

Brain metastases (BM) are the most common brain neoplasm in adults. Current BM therapies still offer limited efficacy and reduced survival outcomes, emphasizing the need for a better understanding of the disease. Herein, we analyzed the transcriptional profile of brain metastasis initiating cells (BMICs) at two distinct stages of the brain metastatic cascade—the “premetastatic” or early stage when they first colonize the brain and the established macrometastatic stage. RNA sequencing was used to obtain the transcriptional profiles of premetastatic and macrometastatic (non-premetastatic) lung, breast, and melanoma BMICs. We identified that lung, breast, and melanoma premetastatic BMICs share a common transcriptomic signature that is distinct from their non-premetastatic counterparts. Importantly, we show that premetastatic BMICs exhibit increased expression of HLA-G, which we further demonstrate functions in an HLA-G/SPAG9/STAT3 axis to promote the establishment of brain metastatic lesions. Our findings suggest that unraveling the molecular landscape of premetastatic BMICs allows for the identification of clinically relevant targets that can possibly inform the development of preventive and/or more efficacious BM therapies.

## Significance

Current treatment options for Brain Metastases (BM) still result in reduced survival outcomes in affected patients, emphasizing the need for a better understanding of the disease and consequently more efficient therapies. In this study, we shed light on the molecular profile of brain metastatic cells during the early stages of the BM cascade and show that targeting certain gene(s) commonly up-regulated in these cells irrespective of their primary tumor of origin is capable of impeding the establishment of BM. Our findings uncover unique factors involved in BM and could lead to the development of more effective and preventive therapeutic options against BM.

Brain metastases (BM) are the most common neoplasm of the central nervous system and a significant cause of cancer-related mortality worldwide. The leading sources of BM are primary lung, breast, and skin (melanoma) cancers (1, 2). However, most cases (~30 to 60%) are reported in lung cancer patients (3), who often simultaneously present with BM at diagnosis (4, 5) Current established treatments for BM, such as surgery and radiotherapy, are seldom effective at fully eradicating the disease, leading to reduced survival times of 4 to 12 mo (3, 5–7) and the need for more effective and targeted therapies.

Experimental models have identified several gene products that aid the outgrowth of metastatic cells in the brain *milieu* (8). However, these models only replicated the later stages of the brain metastatic cascade using bulk tumor cells. Increasing evidence indicates that a small subset of tumor cells with stem-like features termed brain metastasis-initiating cells (BMICs) aid survival in the brain—a site that is typically dissimilar from the primary site of origin of these cells (9). In our previous work, we described the serendipitous capture of lung BMICs at an early or “premetastatic” stage of the BM cascade where BMICs have seeded the brain and started proliferating but have not yet formed visible metastatic lesions (10).

In this study, we report the capture of breast and melanoma BMICs at a similar phase of the BM cascade. We also reveal the transcriptional profiles of premetastatic and non-premetastatic lung, breast, and melanoma BMICs and demonstrate putative brain metastatic functions for HLA-G—one of the genes found to be commonly up-regulated in the premetastatic BMIC cohorts.

## Results

### Capture of Breast and Melanoma BMICs at an Early (Premetastatic) Stage of BM Development.

Less than 0.02% of disseminated tumor cells have the capacity to successfully establish metastases (11). Mounting evidence suggests that this small subset of tumor cells termed metastasis-initiating cells (MICs) exhibit stem-like features that aid survival in a new microenvironment (12). In our previous work, we described the enrichment of stem-like cells from patient-derived lung-BM samples termed lung–brain MICs or lung BMICs that are capable of completing the entire lung-BM cascade (13, 14). Herein, we report using the same methods as described in refs. 13 and 14 and in the *SI Appendix* section, the successful enrichment of stem-like cells from patient-derived breast- and melanoma-BM samples termed parental breast and melanoma BMICs, respectively; hereafter referred to as breast and melanoma BMICs (*SI Appendix*, Table S1). Similar to lung BMICs (13), breast and melanoma BMICs exhibit inherent stem-like features (*SI Appendix*, Fig. S1 *A*–*C*). These BMICs also displayed varying expression levels of the cancer stem cell markers CD44 (15, 16) and CD133 (17, 18) (*SI Appendix*, Fig. S1 *D* and *E*) indicative of interpatient heterogeneity and importantly the need for more defined BMIC markers. Similar to lung BMICs, breast and melanoma BMICs were able to form secondary brain lesions and the xenografts retained the cytoarchitecture and molecular profile of the patient-derived tumors (*SI Appendix*, Fig. S2 *A* and *B* and Table S2).

**Fig. 1.**
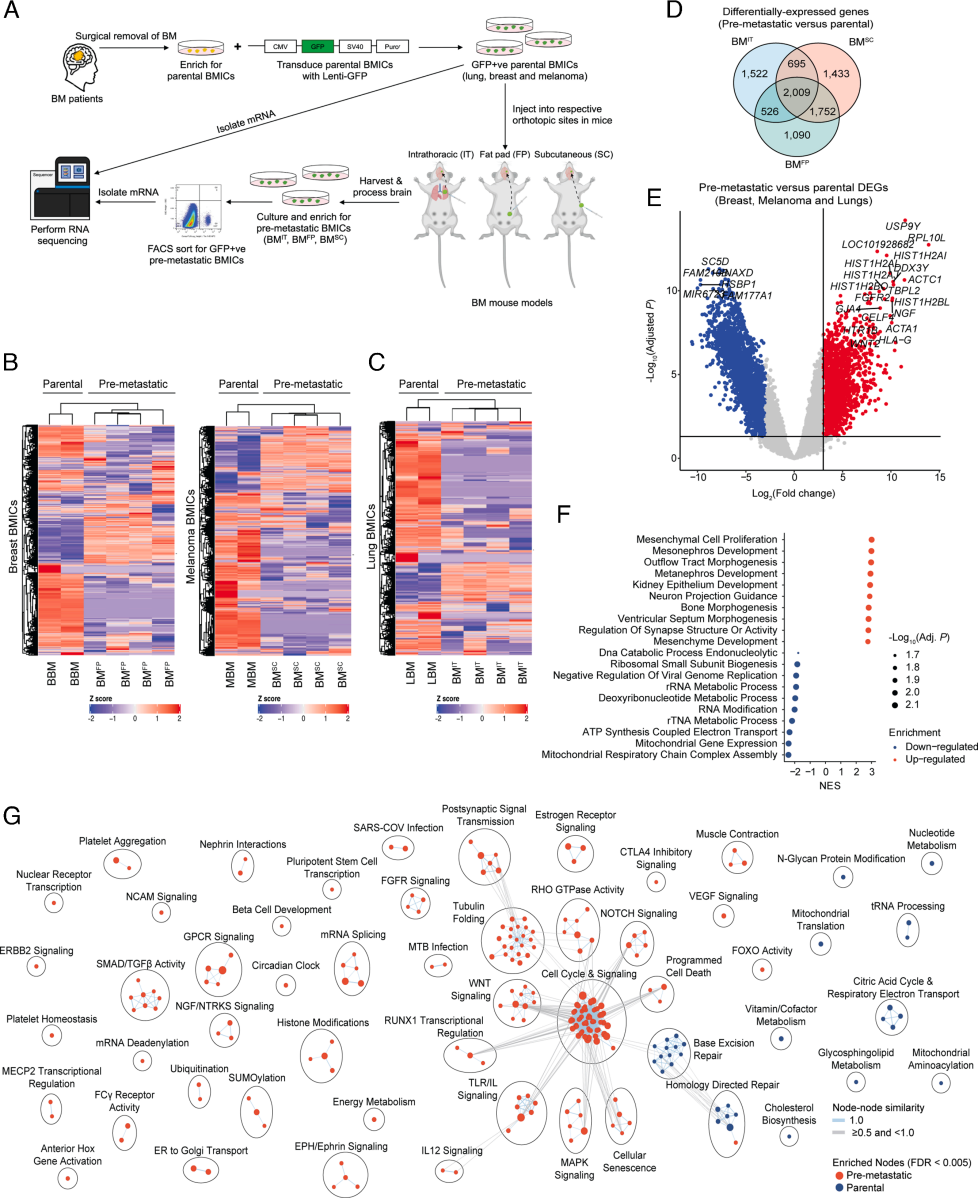
Lung, breast, and melanoma BMICs exhibit a common distinct transcriptomic signature: (*A*) Schematic illustration of premetastatic BMIC model—brain metastatic tumors surgically removed from lung-, breast-, and melanoma-BM patients were processed and cultured in tumorsphere-enriching (NCC- or SCM-supplemented) media (*Methods*) to establish respective patient-derived parental BMICs. Parental BMICs were then tagged with a GFP-expressing vector containing a puromycin-resistant cassette. GFP-tagged parental lung, breast, and melanoma BMICs were subsequently injected into NSG mice via the respective orthotopic routes (lung, fat pad, and subcutaneous). At orthotopic tumor end points, respectively, injected mice were killed, and brains were harvested, processed, and minimally cultured for 2 wk to enrich for premetastatic lung (BM^IT^), breast (BM^FP^), and melanoma (BM^SC^) BMICs, which were then sorted by flow cytometry for GFP positivity. GFP-positive premetastatic and parental BMICs were then subjected to total mRNA isolation and bulk RNA sequencing. Heat map showing the mean expression profile of deregulated genes associated with parental and premetastatic breast, melanoma (*B*), and lung (*C*) BMICs. (*D*) Venn diagram of commonly differentially expressed genes (DEGs) in premetastatic versus parental BMICs. (*E*) Volcano plots showing the commonly up-regulated and down-regulated DEGs in premetastatic breast, melanoma, and lung BMICs. (*F*) Bubble plots showing significantly up- and down-regulated biological processes associated with the differentially expressed gene signature of premetastatic lung, breast, and melanoma BMICs. (*G*) Pathway network showing the different signaling pathways up-regulated and down-regulated in premetastatic lung, breast, and melanoma BMICs. NES – normalized enrichment score.

To capture breast and melanoma BMICs at the premetastatic stage of BM development, we injected early-passage green fluorescent protein (GFP)-tagged parental breast and melanoma BMICs into their respective orthotopic (fat pad and subcutaneous) sites in NOD SCID Gamma (NSG) mice (see *SI Appendix* for detailed information) (Fig. 1*A* and *SI Appendix*, Fig. S3 *A* and *B*). At orthotopic tumor end points, the brains of respective mice groups were harvested, processed, and minimally cultured for 2 wk prior to flow cytometry analysis to capture GFP-positive breast and melanoma BMICs, which yielded a small percentage (~7%) of breast and melanoma BMICs (*SI Appendix*, Fig. S3*C*). We termed these captured breast and melanoma BMIC populations premetastatic because, at this end point/time frame, the corresponding brains of each respective mice group lacked histologically detectable tumor lesions (*SI Appendix*, Fig. S3*C*). Similar to premetastatic lung BMICs (see ref. 10 for detailed information and Fig. 1*A*), premetastatic breast and melanoma BMICs retained their stem-like properties (*SI Appendix*, Fig. S3 *D* and *E*). To study the transcriptomic profiles of premetastatic breast and melanoma BMICs, we isolated total mRNA from these BMICs and their parental (non-premetastatic) BMIC cohorts and subjected the mRNA samples to RNA sequencing (Fig. 1*A*).

### Premetastatic Lung, Breast, and Melanoma BMICs Exhibit a Unique Transcriptomic Profile.

Analysis of our RNA sequencing data revealed that genes from premetastatic breast and melanoma BMICs (Datasets S1 and S2) clustered distinctly from their parental counterparts (Fig. 1*B*), analogous to what we observed in premetastatic lung BMICs (10) (Fig. 1*C* and Dataset S3). Gene set enrichment analysis (19) revealed that differentially expressed genes (DEGs) in premetastatic breast and melanoma BMICs participated in similar biological processes as premetastatic lung BMICs (*SI Appendix*, Fig. S4 and Dataset S4) (10). These remarkable similarities led us to perform combined transcriptomic analyses on normalized datasets of premetastatic lung (GSE110495), breast, and melanoma BMICs and their parental (non-premetastatic) counterparts (*SI Appendix*, Fig. S5) to identify commonly DEGs in the premetastatic cohorts. Notably, we identified ~2,000 genes that are commonly differentially expressed in all premetastatic BMICs independent of their primary tumor of origin (Fig. 1 *D* and *E* and Dataset S5). Gene ontology and pathway analysis revealed biological processes and signaling pathways known to be associated with BM (20–23) including the Wnt, Notch, ERBB2, and NGF/NTRKs pathways (Fig. 1 *F* and *G* and Datasets S6 and S7), highlighting the relevance of the premetastatic gene signature.

### HLA-G Is Highly Expressed in Premetastatic Lung, Breast, and Melanoma BMICs.

**Fig. 2.**
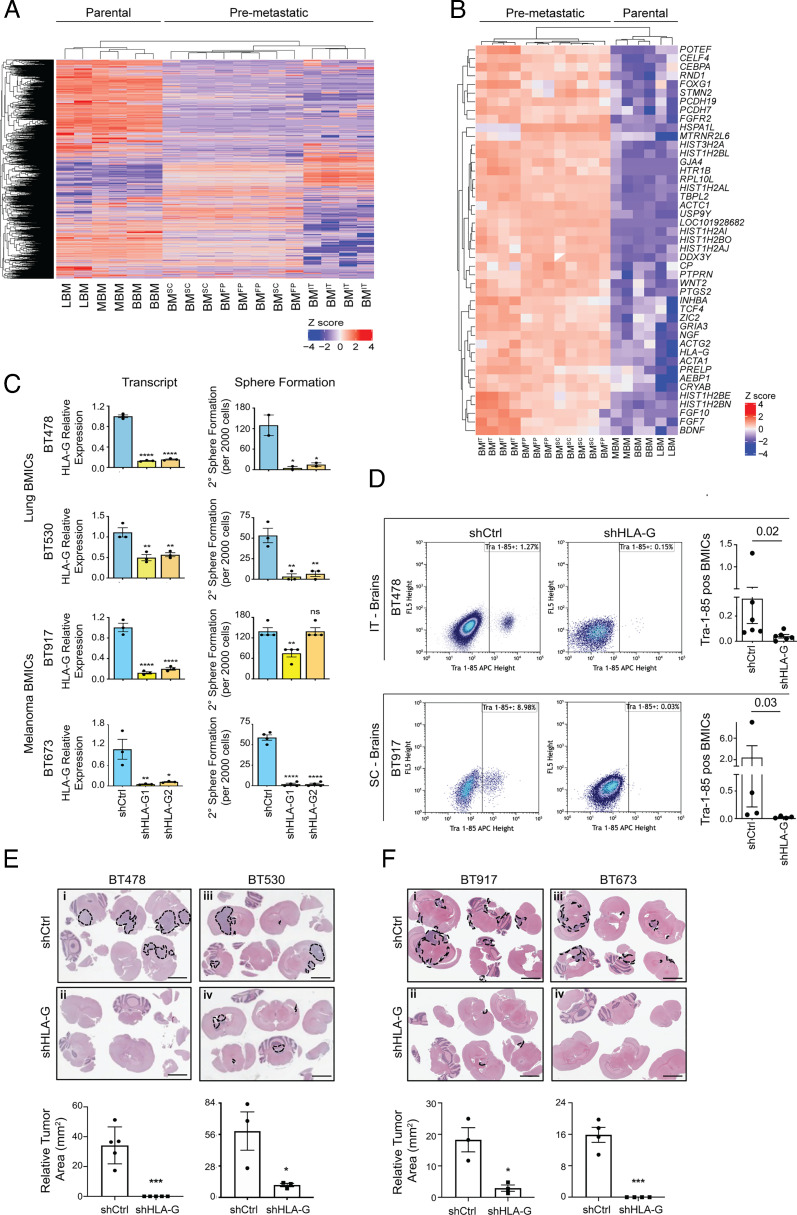
HLA-G knockdown attenuates the establishment of BM: (*A*) Heat map depicting expression of the top commonly up-regulated genes in premetastatic compared to parental BMICs. (*B*) Heat map depicting expression of the top 45 commonly up-regulated genes in premetastatic BMICs. (*C*) Quantitative RT-PCR analysis of HLA-G expression in control (shCtrl) and HLA-G knockdown (shHLA-G1 and shHLA-G2) lung (BT478; BT530) and melanoma (BT917; BT673) BMICs and analysis of HLA-G depletion effects on secondary (2^0^) sphere formation assays in parental lung BMICs. BT478 *P* values for qRT-PCR ****<0.0001 and 2^0^ sphere formation *0.03; BT530 *P* values for qRT-PCR **0.004 and 0.006 and 2^0^ sphere formation **0.002 and 0.002; BT917 *P* values for qRT-PCR **** <0.0001 and 2^0^ sphere formation **0.0040 and ns >0.9999; BT673 *P* values for qRT-PCR **0.0093 and * 0.0126 and 2^0^ sphere formation *P* value **** <0.0001. All experiments were conducted in either duplicate or triplicate. (*D*) Flow cytometry analysis of human Tra-1-85 expression in BMICs enriched from minimally (2 wk) cultured brains of mice intrathoracically (IT) injected with BT478 shCtrl and the most efficient HLA-G knockdown (shHLA-G) BMICs (n = 6 mice each) and subcutaneously (SC) injected with BT917 shCtrl and shHLA-G BMICs (n = 4 mice each). *P* values are shown. To the right are bar graphs depicting % Tra-1-85-positive BMICs captured from the respective brains. (*E*) Hematoxylin and eosin (H&E)-stained images of brain tissues of mice intracranially injected with shCtrl and shHLA-G lung (BT478; BT530) and (*F*) melanoma (BT917; BT673) BMICs at matched time end points. (Scale bar, 500 µm.) Representative images are shown. Below are bar graphs depicting relative tumor areas (mm^2^) of each mouse group. *P* values of shHLA-G tumors with respect to shCtrl tumors are indicated here for BT478 (***0.0003; n = 5 mice), BT530 (*0.049; n = 3 mice), BT917 (*0.02; n = 3 mice), and BT673 (***0.0002; n = 4 mice) mice cohorts. Emboldened black broken lines enclose tumor lesions in each respective mouse brain slice.

We next screened for genes with a threefold or higher expression and an adjusted *P*-value of <0.05 across all the three premetastatic subsets to identify putative genes that may be essential during early-stage BM. We identified 143 genes with consistently higher expression levels in premetastatic BMICs compared to non-premetastatic BMICs (Fig. 2 *A* and *B* and Dataset S8). Notably, five of the candidate genes—*PTGS2* (24, 25), *PCDH7* (24, 26), *INHBA* (13), *ID2* (20, 27), and *TCF4* (20, 28)—have been implicated in late-stage BM, indicating the validity of our screening method, and the added importance of these genes in late-BM stages.

Among the candidate genes was HLA-G, a well-established tolerogenic molecule (29) (Fig. 2*B*), which was surprising as the in vivo BM models (NSG mice) used in this study are severely immunocompromised. This piqued our interest and led us to investigate the plausible nonimmune-related brain metastatic roles of HLA-G in early-stage BM. Notably, HLA-G has also been recently shown to be up-regulated in advanced lung–brain metastatic tumors when compared to their primary counterparts (30).

In line with our RNA sequencing data, we observed significantly higher HLA-G expression at both the transcript and protein levels in premetastatic lung BMICs compared to parental controls (*SI Appendix*, Fig. S6 *A* and *B*). As we were only able to establish premetastatic lung BMIC lines, we could not perform similar validation experiments in premetastatic breast and melanoma BMICs. Nonetheless, the validated increase in HLA-G expression in premetastatic lung BMICs indicated a prospective role for HLA-G in early-stage BM.

### HLA-G Knockdown Attenuates BM Establishment.

To investigate HLA-G functions in early-stage BM, its basal expression was first depleted in parental lung and melanoma BMICs using HLA-G-specific shRNAs. All further in vitro and in vivo experiments in this study were only performed in parental lung and/or melanoma BMICs due to our inability to establish parental breast BMIC lines from the BMICs enriched from patient breast–brain metastatic tumors (*SI Appendix*, Fig. S1*A* and Table S1). Functional analysis of control (shCtrl) and HLA-G depleted (shHLA-G1 and 2) parental lung and melanoma BMICs in vitro revealed a reduction in the secondary sphere formation abilities of lung and melanoma BMICs upon HLA-G knockdown (Fig. 2*C*). Remarkably, HLA-G is affiliated with multicellular sphere formation in cancer cells in vitro (31) implying a role for HLA-G in cancer stem cells self-renewal. We also observed a reduction in the proliferation of lung and melanoma BMICs upon HLA-G knockdown 4-d postseeding (*SI Appendix*, Fig. S7*A*), indicating a role for HLA-G in tumor cell proliferation that has also been reported elsewhere (32, 33).

Next, we performed in vivo studies using orthotopic BM models injected with shCtrl or the most efficient shHLA-G lung and melanoma BMICs selected for 2 d posttransduction (*SI Appendix*, Fig. S7*B*). We found that HLA-G depletion did not result in any significant difference in the timeframe to orthotopic lung (*SI Appendix*, Fig. S8*A*; n = 6, *P* = 0.59) and melanoma (*SI Appendix*, Fig. S8*B*; n = 4; *P* = 0.53) tumor end points nor did it affect the ability of lung and melanoma BMICs to form orthotopic tumors (*SI Appendix*, Fig. S8 *C* and *D*; n = 5; *P* = 0.89, and n = 4; *P* = 0.66, respectively). However, HLA-G depletion greatly inhibited the capacity of lung and melanoma BMICs to accumulate in the brain. This was demonstrated by the low percentage of Tra-1-85-positive HLA-G-depleted lung (0.00 to 0.15%) and melanoma BMICs (0.01 to 0.03%) compared to the high percentage of Tra-1-85-positive control lung (0.03 to 1.27%) and melanoma (0.03 to 8.98%) BMICs captured from the processed brains of the respective mice groups (Fig. 2*D*; n = 6, *P* = 0.02, and n = 4; *P* = 0.03). These findings indicated an important role for HLA-G during early-stage BM when brain metastatic cells colonize and accumulate in the brain parenchyma to establish micro- and macro-metastatic brain lesions (34).

Since orthotopic BM models do not allow the visualization of late-stage macrometastatic BM, we directly injected control and HLA-G-depleted parental lung and melanoma BMICs into the brains of NSG mice to investigate HLA-G roles in the ability of brain metastatic cells to establish mature brain lesions. We noted that HLA-G loss attenuated the ability of lung and melanoma BMICs to form macrometastatic brain lesions compared to the large tumors formed by their time-matched controls (Fig. 2 *E*, *i* and *ii*, n = 5, *P* = 0.0003 and Fig. 2 *E*, *iii* and *iv*, n = 3, *P* = 0.049; Fig. 2 *F*, *i* and *ii*, n = 3; *P* = 0.02 and Fig. 2 *F*, *iii* and *iv*, n = 4; *P* = 0.0002, respectively). These findings suggested that HLA-G is also required for brain metastatic cells to thrive in the brain and form mature brain lesions.

### HLA-G Promotes BMICs’ Stem-Like Traits and Growth in the Brain Milieu via STAT3 Signaling.

**Fig. 3.**
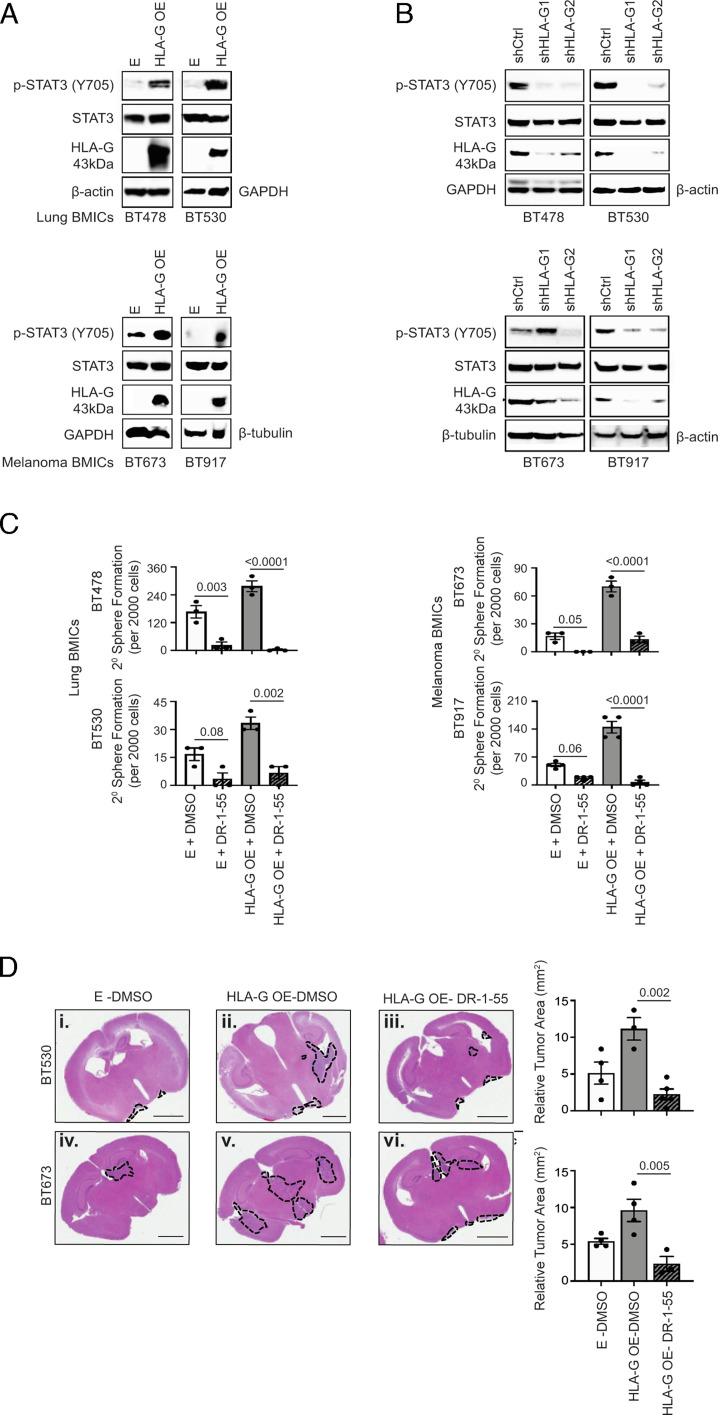
HLA-G promotes BMICs’ secondary sphere formation and growth in the brain parenchyma via STAT3 signaling: (*A*) Western blot analysis of HLA-G (low exposure), pSTAT3 (Y705) and STAT3 expression in control (E), and HLA-G OE lung (BT478; BT530) and melanoma (BT673; BT917) BMICs; 6 µg of protein was used, and (*B*) control (shCtrl) and HLA-G knockdown (shHLA-G1 and shHLA-G2) lung and melanoma BMICs; 15 µg of protein was used with GAPDH, β-actin, and β-tubulin serving as loading controls. (*C*) In vitro characterization (2^0^ sphere formation assays) of vehicle-treated (DMSO) and DR-1-55-treated E and HLA-G OE lung (BT478; BT530) and melanoma (BT673; BT917) BMICs. *P* values are shown in bar graphs. All experiments were conducted in either duplicate or triplicate. (*D*) Hematoxylin and eosin (H&E)-stained images of brain tissues of mice intracranially injected with vehicle (DMSO)-treated control (E-DMSO and HLA-G OE-DMSO) and DR-1-55-treated HLA-G OE (HLA-G OE DR-1-55) lung (BT478) and melanoma (BT673) BMICs at matched time end points. Emboldened black broken lines enclose tumor lesions in each respective mouse’s brain. To the right are bar graphs depicting the relative tumor areas (mm2) of each mouse group. *P* values are indicated. Representative images are shown. (Scale bar, 2 mm.)

To determine how HLA-G promotes BMICs stem-like features and growth in the brain as described in Fig. 2 *C*–*F*, we overexpressed HLA-G in parental lung BMICs. Functional characterization of HLA-G overexpressing (HLA-G OE) lung BMICs showed that HLA-G localized to both the cytoplasm and cell membrane (*SI Appendix*, Fig. S9 *A* and *B*). HLA-G overexpression increased the secondary sphere formation of lung BMICs in vitro (*SI Appendix*, Fig. S9*C*; *P* = 0.03) and boosted their growth in vivo (*SI Appendix*, Fig. S9*D*; n = 5; *P* = 0.048). These findings complemented our HLA-G depletion studies and validated a role for HLA-G in BMIC self-renewal and establishment in the brain. We next evaluated the effect of HLA-G overexpression on downstream effectors of STAT3 [phospho(p)-STAT3 (Tyr705)], ERK (p-ERK1/2 Thr202/Tyr204), and AKT [p-AKT (Ser473)] signaling cascades since these pathways are implicated in the regulation/maintenance of normal/cancer stem cells (35–37) and BM establishment (38–40) and are also demonstrated in other studies to be activated by membrane HLA-G (33, 41). Interestingly, we observed increased levels of p-STAT3 (Fig. 3*A*), but reduced levels of p-ERK1/2 and p-AKT in response to HLA-G overexpression in lung BMICs (*SI Appendix*, Fig. S9*E*), indicating that high HLA-G levels stimulate STAT3 but not ERK and AKT signaling in lung BMICs. We also observed increased p-STAT3 levels in melanoma BMICs upon HLA-G overexpression (Fig. 3*A*), signifying that HLA-G activation of STAT3 signaling is not restricted only to lung BMICs. Complementary studies in control and HLA-G-depleted parental lung and melanoma BMICs showed that HLA-G ablation resulted in reduced p-STAT3 levels in lung and melanoma BMICs when compared to their control counterparts (Fig. 3*B*), demonstrating a bona fide role for HLA-G in STAT3 signaling activation in BMICs.

To determine whether HLA-G promotes BMIC self-renewal and growth in the brain through STAT3 signaling, we inhibited STAT3 signaling in HLA-G OE lung and melanoma BMICs with the STAT3 inhibitory drug DR-1-55 (42) (See *SI Appendix*, Fig. S10 *A* and *B* for IC_50_ values). Intriguingly, suppression of STAT3 signaling with DR-1-55 in HLA-G OE BMICs (*SI Appendix*, Fig. S10*C*) caused a drastic decline in the secondary sphere formation of HLA-G OE lung and melanoma BMICs despite the presence of high HLA-G levels in these cells (Fig. 3*C*). We also saw a significant reduction in brain tumor lesions formed by HLA-G OE lung and melanoma BMICs upon STAT3 signaling inhibition ex vivo (Fig. 3 *D*,  *i*–*iii*; *P* = 0.002; Fig. 3 *D*,  *iv*–*vi*; *P* = 0.005) despite the overexpressed HLA-G levels in these cells. Our findings indicate that HLA-G functions through the STAT3 pathway to enhance BMIC stem-like traits and growth in the brain.

### HLA-G Stimulates STAT3 Signaling in BMICs via SPAG9.

**Fig. 4.**
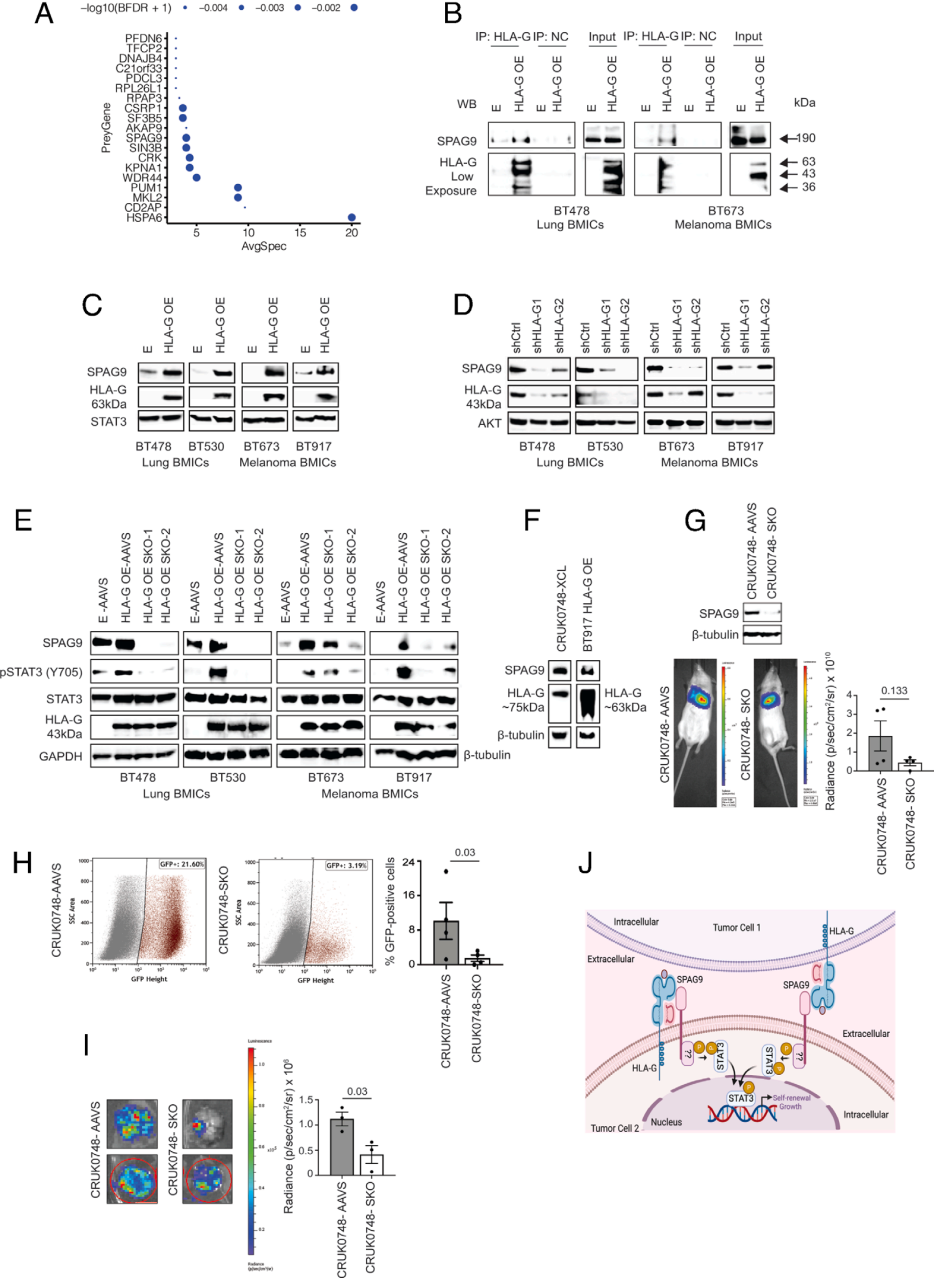
HLA-G stimulates STAT3 signaling via SPAG9 in BMICs and targeting SPAG9 in brain-tropic cells attenuates BM: (*A*) Bubble plot showing HLA-G-interacting proteome in parental lung (BT478) BMICs. (*B*) Immunoprecipitation (IP) experiments demonstrating that HLA-G binds SPAG9 in control (E) and HLA-G OE lung (BT478) and melanoma (BT673) BMICs. WB – Western blot; NC – Negative control. Western blot analysis of SPAG9 and HLA-G expression in *E* and HLA-G OE lung (BT478; BT530) and melanoma (BT673; BT917) BMICs – 6 µg of protein was used and HLA-G shown is at a low exposure (*C*) and control (shCtrl) as well as HLA-G knockdown (shHLA-G1 and shHLA-G2) lung and melanoma BMICs—~15 µg or 20 µg of protein was used and the exposure of HLA-G here is higher. (*D*) STAT3 and AKT serve as loading controls in the respective western blots. (*E*) Western blot analysis of SPAG9, pSTAT3 (Y705), STAT3, and HLA-G expression in control (E-AAVS and HLA-G OE-AAVS) and HLA-G OE pooled SPAG9 knockout (HLA-G OE SKO-1 and SKO-2) lung and melanoma BMICs, with GAPDH and β-tubulin serving as loading controls. 8 µg of protein was used and HLA-G shown is at a low exposure. All experiments were conducted in either duplicate or triplicate. (*F*) Western blot analysis of SPAG9 and HLA-G expression in a patient-derived primary lung cancer cell line (CRUK0748-XCL) derived from a subcutaneous xenograft (PDX) of the lung tumor. 10 µg of protein was used. BT917 HLA-G OE BMICs serves as a positive control for HLA-G expression, while β-tubulin serves as a loading control. HLA-G ~63 kDa and ~75 kDa represents the dimeric form, while HLA-G 43 kDa represents the monomeric form of the membrane HLA-G isoform 1 (HLA-G1) expressed in the respective cells as indicated, see ref. 48 for reference. (*G*) Western blot analysis of SPAG9 expression in control (CRUK0748-XCL-AAVS) and the most efficient pooled SPAG9 knockout (CRUK0748-XCL-SKO) in patient-derived primary lung cancer cells (CRUK0748-XCL). β-tubulin serves as a loading control. Beneath is in vivo imaging system (PerkinElmer) used to acquire bioluminescence images of lung tumors formed by CRUK0748-XCL-AAVS and CRUK0748-XCL-SKO cells injected into the lung [intrathoracically (IT)] of NSG mice (n = 4 each). To the right is a bar graph depicting the radiance values of the lung tumor lesions. *P* value is indicated. (*H*) Flow cytometry analysis of GFP-positive CRUK0748-XCL cells isolated from minimally (2 wk) cultured brains of mice IT injected with CRUK0748-XCL-AAVS and CRUK0748-XCL-SKO cells (n = 4 mice each). To the right is the bar graph depicting % GFP-positive cells captured from the respective brains. *P* value is shown. (*I*) Bioluminescence images of brain lesions formed by CRUK0748-XCL-AAVS and CRUK0748-XCL-SKO cells injected into NSG mice IT (n = 3; no signal was obtained from the 4th mice brain set, which corresponds to the least % of cells captured from the respective mice brains—see *E*). To the right is a bar graph depicting the radiance values of the brain lesions. The *P* value is indicated. (*J*) Schematic illustration of the potential role of HLA-G and SPAG9 in STAT3 signaling. HLA-G interacts with SPAG9 either on the same tumor cell or an adjacent tumor cell. This interaction leads to the phosphorylation of STAT3 by a yet unidentified kinase, which then promotes BMICs’ self-renewal (secondary sphere formation) abilities and growth. Created with BioRender.com.

HLA-G is known to activate STAT3 signaling in noncancerous cells particularly dendritic and myeloid cells through its interaction with ILT4 (43, 44). However, in cancer cells, it is unknown whether HLA-G activates STAT3 pathway via ILT4 or other yet unidentified protein partners. When we did not find high expression of known HLA-G partners such as ILT4 (*LILRB2*) or ILT2 (*LILRB1*) in our lung BMIC transcriptomic dataset—GSE110495 (10) (*SI Appendix*, Fig. S11), we postulated that HLA-G stimulated STAT3 signaling in BMICs *via* other unknown protein partners. Through BioID experiments, we unraveled 10 proteins proximal to HLA-G in BMICs with no currently known HLA-G protein partners (ILT4, ILT2, or KIR2DL4) identified in our list (Fig. 4*A* and Dataset S9). Since membrane HLA-G expression is linked with STAT3 activation in cancer cells (41), we chose the membrane localizing SPAG9 protein (45) from the candidate HLA-G protein partners for further investigation. Immunoprecipitation studies confirmed the association of HLA-G with SPAG9 in lung and melanoma BMICs (Fig. 4*B*). Surprisingly, we noticed an increase in SPAG9 protein levels in HLA-G OE lung and melanoma BMICs compared to their respective controls (Fig. 4*C*). We also observed reduced SPAG9 expression in HLA-G-depleted lung and melanoma BMICs (Fig. 4*D*), indicating a positive correlative relationship between HLA-G and SPAG9 in BMICs. In support of these results, other studies have observed a positive relationship between HLA-G and its established protein partners—ILT2, ILT4, and KIR2DL4 (46) where HLA-G has been shown to particularly up-regulate ILT4 expression in tumor cells when stimulating cell signaling pathways (33, 47).

To elucidate whether HLA-G stimulates STAT3 signaling in BMICs via SPAG9, we generated pooled *SPAG9* knockout in HLA-G OE lung BMICs and probed for the expression of p-STAT3 (Y705) in control and *SPAG9* knockout lung and melanoma BMICs. Pooled HLA-G OE *SPAG9* knockout cells did not have complete *SPAG9* deletion (Fig. 4*E*). Nonetheless, we found that SPAG9 ablation in HLA-G OE lung and melanoma BMICs diminished the expression of p-STAT3 despite the high HLA-G levels present in these cells (Fig. 4*E*), demonstrating that HLA-G modulates STAT3 signaling in BMICs via SPAG9.

### Targeting SPAG9 in Primary Lung Cancer Cells Is Capable of Preventing BM.

High HLA-G and SPAG9 expression has been independently linked to poor survival outcomes in lung and other cancers (49–51). However, it is not known whether high levels of HLA-G and/or SPAG9 in primary cancers could predict patients at risk of developing BM. Unfortunately, to our knowledge, there are no publicly available datasets with gene expression data from primary lung tumors and clinical annotation regarding their matched BM that we can use to investigate the clinical relevance of high *HLA-G* and *SPAG9* expression on the formation of BM.

As an alternative approach to determine the clinical significance of *HLA-G* and *SPAG9* on BM predisposition in primary lung tumors, we queried gene chip and RNA-seq lung tumor datasets through the KMplotter portal (52, 53) to determine whether *HLA-G* and *SPAG9* expression in primary lung tumors predicts poor patient survival; since, a large percentage (20 to 50%) of lung cancer patients develop BM, which correlates with limited survival in affected patients (54). We found that lung cancer patients with either high *HLA-G* or *SPAG9* tumors, or a combination of both high *HLA-G* and *SPAG9* expressing tumors, exhibited poor overall survival compared to their low *HLA-G-* and *SPAG9*-expressing tumor counterparts (*SI Appendix*, Fig. S12 *A*–*F*), with high *SPAG9*-expressing tumors yielding the most significant effect on overall survival in the two datasets analyzed (*SI Appendix*, Fig. S12 *B* and *E*).

Thus, we next explored the possibility of preventing BM by targeting SPAG9 in primary cancers with a high predilection for BM. Because lung cancers account for a higher proportion (~30 to 60%) of BM (3), we used primary lung cancer cells for this experiment and established a unique xenograft-derived cell line from a patient who was known to have developed lung-to-BM (CRUK0748-XCL). Analysis of SPAG9 and HLA-G expression in parental CRUK0748-XCL cells revealed that both SPAG9 and HLA-G are expressed in parental CRUK0748-XCL, signifying that HLA-G and SPAG9 are expressed in primary lung cancer cells with a high predisposition to BM. Notably, HLA-G was seen expressed at a higher molecular weight band (~75 kDa), representing the dimeric form of HLA-G1 (see ref. 48 for reference) (Fig. 4*F*).

For in vivo experimentation, we generated *SPAG9* knockout in GFP- and luciferase-expressing parental CRUK0748-XCL cells. Control or the most efficient pooled *SPAG9* knockout CRUK0748-XCL cells (Fig. 4*G*) were then orthotopically injected into the lungs of NSG mice. At orthotopic tumor end points, we observed that the lung tumor burden of the *SPAG* knockout mice group was slightly reduced compared to controls, but this decrease was not statistically significant (Fig. 4*G*). Flow cytometry analysis for GFP-positive CRUK0748-XCL cells in minimally cultured brains from control and *SPAG9* knockout mice groups revealed a statistically significant reduction in the percentage of GFP-positive CRUK0748-XCL cells captured from the brains of the *SPAG9* knockout mice (Fig. 4*H*). This result corresponded to the significant decrease in brain lesions noted in the *SPAG9* knockout mice as ascertained by in vivo imaging (Fig. 4*I*).

Our findings demonstrate that targeting *SPAG9* mitigates the ability of primary lung cancer cells to accumulate in the brain and establish mature brain lesions. This observation is significant because it hints that in a preventive setting, SPAG9 ablation in primary lung tumor cells is sufficient to inhibit BM despite the presence of HLA-G in these cells, which indicates an important role for SPAG9 in HLA-G’s ability to promote BM.

## Discussion

In this study, we have uncovered gene products involved in the early stages of the brain metastatic cascade and show that despite the primary tumor of origin, brain metastatic cells exhibit a common transcriptomic signature at early-BM stages that diversify at later-BM stages. This suggests a uniform but temporally dynamic molecular programming in cancer cells as they first encounter the brain microenvironment. Our data thus provide a significant resource that can be mined for biomarkers to serve as candidates for diagnostic and drug development/treatment approaches against BM. Correspondingly, we identified a nonimmune-related role for one of the top candidate genes (HLA-G) in BM establishment. Since HLA-G is a well-established immunosuppressive and immunomodulatory molecule in multiple cancers including glioblastoma (29, 55–58), we do not rule out that it may also have immune-related functions in BM, which has recently been suggested by (30). Thus, though beyond the scope of this study, further investigation is needed in immunocompetent BM models to understand the full roles of HLA-G in BM.

Another unique finding in this work is the identification of an HLA-G protein partner—SPAG9. It has been postulated that HLA-G may interact with unknown protein partners in tumor cells to activate the STAT3 pathway (41), but this study actually identifies a contemporary HLA-G binding partner with respect to its activation of the STAT3 pathway. Moreover, we demonstrate that targeting SPAG9, which is expressed together with HLA-G in primary lung cancer cells, is sufficient to impede BM establishment despite the presence of HLA-G in these cells, signifying a potential preventive therapeutic role for an HLA-G intermediary partner in primary cancers with a predilection for BM. It is noteworthy to mention that we also identified other putative protein partners of HLA-G through our BioID screen that warrant further investigation to provide a more comprehensive knowledge of HLA-G’s role in BMICs and BM formation and, putatively, additional viable therapeutic targets in BM.

## Conclusion

Collectively, our findings provide clinically relevant insights into the transcriptional profile of malignant cells during the early phase of the brain metastatic cascade before these cells evolve tumor-specific properties. Such data will hopefully lead to therapies that target highly brain-specific malignant cells early on in the brain metastatic process before they fully colonize the brain, which could potentially prevent BM and increase survival outcomes in at-risk patients while preserving their quality of life and neurocognitive function (16, 17).

## Methods

### Patient Samples.

Human lung–brain, breast–brain, and melanoma-brain metastases patients’ samples were obtained from Hamilton Health Sciences with written consent from patients as approved by the Hamilton Health Sciences/McMaster Health Sciences research ethics board (REB #07366), in compliance with Canada’s Tri-Council policy statement on the ethical conduct for research involving humans and International ethical guidelines for biomedical research involving human subjects. Patient-derived samples were deidentified before use.

### Cell Culture.

Human lung–brain, breast–brain, and melanoma-brain metastases patients’ samples were used to derive lung-, breast-, and melanoma-BMICs, while human primary lung cancer samples were used to generate the xenograft-derived CRUK0748-XCL primary lung cancer cell line. Please see *SI Appendix* for more information on culture conditions for the respective lines.

Control and HLA-G-overexpressing lung BMICs were treated with vehicle (DMSO) and IC_80_ values (see *SI Appendix*, Fig. S10 *A* and *B* for IC_50_ values) of DR-1-55 (a kind gift from Dr. Patrick Gunning; Department of Chemistry, University of Toronto, Canada) for 4 d for secondary sphere formation assays and 3 d for ex vivo experiments.

Please see *SI Appendix* for all other methods description.

## Supplementary Material

Appendix 01 (PDF)

Dataset S01 (XLS)

Dataset S02 (XLS)

Dataset S03 (XLS)

Dataset S04 (XLS)

Dataset S05 (XLS)

Dataset S06 (XLS)

Dataset S07 (XLS)

Dataset S08 (XLSX)

Dataset S09 (XLSX)

Dataset S10 (XLSX)

Dataset S11 (XLSX)

## Data Availability

The lung RNA-Seq data discussed in this publication have been deposited in NCBI’s Gene Expression Omnibus and are accessible through GEO Series accession number GSE110495 (https://www.ncbi.nlm.nih.gov/geo/query/acc.cgi?acc1/4GSE110495). The processed data obtained from the breast and melanoma RNA-Seq dataset, which were used in this study, have been included as *SI Appendix* and Datasets S1, S2, S5, and S8 in this study. The raw and processed RNA sequencing data for the breast and melanoma BMICs have also been deposited in NCBI’s Gene Expression Omnibus and are accessible through GEO Series accession number GSE220156.
