# Supplementary material for: An HLA-G/SPAG9/STAT3 axis promotes brain metastases
Source: Proc Natl Acad Sci U S A. 2023 Feb 13;120(8):e2205247120. doi: 10.1073/pnas.2205247120 (PMC9974476; doi:10.1073/pnas.2205247120)
Supplement: Supplementary file 1 — Appendix 01 (PDF) [file pnas.2205247120.sapp.pdf]

Materials and Methods

Cell Culture

Human lung-brain, breast-brain and melanoma-brain metastases patients' samples were used to derive lung, breast and melanoma-brain metastasis initiating cells (BMICs) while human primary lung cancer samples were used to generate the xenograft-derived primary lung cancer cell line. The lung BMICs (BT478 and BT530) were cultured in NeuroCult Complete (NCC) media consisting of NeuroCultTM NS-A Basal Medium (#05750; StemcellTM Technologies, Vancouver, BC, Canada) complemented with 50 mL of NeuroCultTM Supplement (#05751; StemcellTM Technologies), 20ng/mL human recombinant epidermal growth factor (EGF) (#78006.2; StemcellTM Technologies), 10ng/mL human recombinant fibroblast growth factor (FGF) (#78003.2; StemcellTM Technologies), 0.2% heparin (#07980; StemcellTM Technologies) and 1% antibiotic-antimycotic solution (#450-115-EL; Wisent Inc., Saint-Jean-Baptiste, QC, Canada). Breast (BT922, BT923, BT930, MBT08 and MBT65) and melanoma (BT925, BT969, BT917 and BT673) BMICs were cultured in stem cell media (SCM) consisting of Dulbecco's Modified Eagle Medium (DMEM)/F12 GlutaMAX™ media (#10565018; Thermo Fisher Scientific, Waltham, MA, U.S.A.) supplemented with 0.2% 50X B27 supplement (#17504044; Gibco, Gaithersburg, MD 20877, U.S.A.), 20ng/mL human recombinant epidermal growth factor (EGF) (#78006.2; StemcellTM Technologies), 10ng/mL human recombinant fibroblast growth factor (FGF) (#78003.2; StemcellTM Technologies), 0.2% heparin (#07980; StemcellTM Technologies) and 1% antibiotic-antimycotic solution (#450115-EL; Wisent Inc.). Human fetal neural stem cells (hNSCs) were isolated using a previously described protocol (78) and cultured in NCC-supplemented media. Normal human astrocytes (NHA) were purchased from American Type Culture Collection (Manassas, VA, USA) while CRUK0748-XCL cultures were established from a fourth passaged subcutaneous xenograft derived from a patient with lung adenocarcinoma. Both NHA and CRUK0748-XCL were cultured in DMEM/F12 media (#10565018; Thermo Fisher Scientific) supplemented with 10% fetal bovine serum (FBS; # 080-450; Wisent Inc.) and 1% antibiotic-antimycotic solution. All BMICs and hNSC lines were grown as tumorspheres while NHA and the CRUK0748-XCL were cultured adherently prior to conduct of experiments and maintained at 37ºC with a humidified atmosphere of 5% CO2.

BMIC lines were dissociated into single cell suspension prior to experimental assays using Liberase (#05401119001; Roche, Mannheim, Germany) for cells grown in suspension or TrypLE (#12605028; Thermo Fisher) for adherent cells.

Generation of pre-metastatic breast and melanoma BMICs

Patient-derived parental GFP-tagged breast and melanoma BMICs were injected into their respective orthotopic (fat pad and subcutaneous) sites in NSG mice (n = 2 mice each). At orthotopic tumor endpoints (tumor volume of ~100-500mm^3; **SI Appendix, Figs. S3a-b)**, the brains were harvested, processed, and minimally cultured for 2 weeks prior to flow cytometry analysis of GFP-positive BMICs, which yielded a small percentage (~7%) of breast and melanoma BMICs respectively from the processed brains of each mice group (**SI Appendix, Figs. S3c**). We categorized these BMICs as pre-metastatic breast and melanoma BMICs, because the respective mice brains lacked histologically detectable tumor lesions (**SI Appendix, Figs. S3c**)

RNA Sequencing

Illumina sequencing was performed as previously described (15) on bulk RNA isolated from minimally (2 weeks) cultured pre-metastatic breast and melanoma BMICs and their parental BMIC cohorts. Raw RNA sequencing data were processed and normalized as also described (15). We chose to use parental breast and melanoma BMICs as our non-pre-metastatic controls since our initial studies with lung BMICs had demonstrated a similar genetic profile between parental (i.e., established or macro-metastatic) lung BMICs and lung BMICs grown orthotopically in mice (1).

Principal component and differential expression analyses

Principal component analysis (PCA) was performed on normalized counts per million (CPM) values of all samples using edgeR (79) (v3.30.3) and stats (v4.0.4). Normalization was performed using calcNormFactors (method = 'TMM') and log2-transformed using voom(). Differentially expressed genes (DEGs) were identified between indicated cohorts using the Bioconducter packages limma (80) (v3.44.3) and edgeR (79) (v3.30.3).

The read count matrix was filtered to remove low-expressed genes using the function filterByExpr() using default parameters. A linear model was fit to account for differences between pre-metastatic (BMIT/SC/FP) and parental (LBM, BBM, MBM) samples. DEGs were extracted using treat() (limma) and determined using log2(fold change)>1 and adjusted $p<0.05$.

Gene Set Enrichment Analysis

Indicated differential expression analysis of pre-metastatic (BMIT/SC/FP) and parental (LBM, BBM, MBM) samples were used to conduct gene set enrichment analysis (GSEA) (29). Gene sets belonging to GO biological processes (c5.go.bp.v7.2.symbols) and reactome pathways (c2.cp.reactome.v7.2.symbols) were obtained from MSigDB Collection (www.gsea-msigdb.org). GSEA was conducted with a minimum of 15 and maximum of 300 genes in each gene set, a total of 10,000 permutations, and an adjusted $p<0.05$. GSEA results were visualized using Cytoscape (v3.8.0) and processed using Enrichment Map (v3.3.0) and AutoAnnotate (c1.3.3) functions to determine themes of overlapping gene sets.

Quantitative Reverse Transcription and Polymerase Chain Reaction (qRT-PCR)

Whole mRNA was isolated from BMICs using the Norgen RNA extraction kit (#37500; Biotek, Thorold, ON, Canada) and then reverse transcribed into complementary DNA (cDNA) with the iScript™ Reverse Transcription Super mix for RT-qPCR (#1708840; BioRad, Mississauga, ON, Canada) and the C1000 Thermo Cycler (Bio-Rad) according to the manufacturer's instructions. Quantitative PCR was subsequently performed using the SYBR qPCR mix (#A25742; Applied Biosystems, Burlington, ON, Canada), gene-specific primers (**SI Appendix, Table S3**; primers were generated by IDT Technologies using standard desalting techniques according to manufacturer's protocols) and the ribosomal protein 28S as the internal control (as we observed no variance between samples using this control) in the following master mix reaction volume for 1 reaction that was set up manually – PowerUp SYBR Green Master mix – 5uL, 10uM forward and reverse primers – 0.5uL each, RNAse/DNAse free water – 2uL and 400ng of cDNA – diluted to ensure corresponds to 2uL per sample to give a total of 10uL placed in a clear 96-well multiplate® PCR plates (#MLL9601; Bio-RAD,

Mexico). Reaction volume was scaled up as needed per reaction with a minimum of duplicate and maximum of triplicate wells for each sample. In the no template reaction wells, cDNA was replaced by 2uL of RNAse/DNAse free water. Reaction was run on the QuantStudio™ 3 Real-Time PCR System, 96-well, 0.2 mL (#A28567; Thermo Fisher Scientific) using the following protocol – lid – 105°C, hold stage - 95°C for 30 seconds, PCR stage step 1 - 95°C for 5 seconds, step 2 - 64°C for 30 seconds, step 3 72°C for 30 seconds repeated 39 times and a continuous melt curve stage at step 1 - 95°C for 30 seconds, step 2 - 65°C for 5 seconds, step 3 - 95°C for 15 seconds. Results were analyzed using the Thermo Fisher Scientific qPCR software affiliated with the instrument ((#A28567) and illustrated and assessed for significance using the GraphPad Prism 6 software with statistical significance as described in the statistics session. Information has been provided above as much as possible according to the MIQE guidelines (2).

Western blot analysis

Total protein was isolated from BMICs with 1X RIPA buffer and denatured protein resolved on a NuPAGE™ 4 - 12%, Bis-Tris, 1.5 mm, 10-well mini protein gel (#NP0335BOX; Invitrogen™). Resolved proteins were then transferred onto polyvinylidene difluoride (PVDF) membranes, and membranes blocked for 30 minutes at room temperature with either 3% bovine serum albumin (BSA; #PI23209; Thermo Scientific™ Pierce™) in 1X Tris-buffered saline (TBS)-0.05% Tween-20 for phosphorylated proteins or 5% skimmed dry milk in 1X TBS-Tween 20 (TBS-T). Blocking was followed by incubation with primary antibodies (**Dataset S10**) as well as a loading control of interest (**Dataset S10**) at 4°C overnight. Following primary antibody incubation, membranes were washed in 1X TBS-T 5 times for 5 minutes before subsequent incubation with a goat anti-mouse horseradish peroxidase (HRP)-conjugated (#170-6516; BioRad; 1:5000) or a goat anti-rabbit HRP-conjugated secondary antibody (#170-6515; BioRad; 1:5000) for one hour at room temperature. Band visualization was performed using LuminataTM Forte Western HRP Substrate (#WBLUF05000; Millipore-Sigma, St. Louis, MO, U.S.A.) and protein detection using the BioRad Chemidoc MP (Serial No: 731BR01445). Immunoblots were quantified and normalized to the loading control with ImageJ software.

Immunocytochemistry

600,000 cells were plated directly onto clean cover slips (12x12mm) placed in the bottom of each well of a 12-well plate and incubated in NCC complete media supplemented with 10% fetal bovine serum (FBS) for 24 hours at 37°C with a humidified atmosphere of 5% CO2. Once cells had adhered on cover slips, they were washed twice with 1X PBS, then fixed with ice cold methanol for 10 minutes at -20°C. After fixation, adhered cells were washed twice with 1X PBS and then non-specific binding blocked with 3% skimmed milk in PBS for 30 minutes at room temperature. Once blocking was done, cells were incubated with mouse monoclonal anti-HLA-G antibody (4H84; sc-21799; 1:30; Santa Cruz Biotechnology, Inc., Dallas, Texas, U.S.A.) for two hours at room temperature. Following incubation, cells were washed twice with 1X PBS and then incubated in Alexa Fluor® 488 goat anti-mouse secondary antibody (Invitrogen- Life Technologies Corporation, Eugene, OR, U.S.A.; A11029) for one hour at room temperature in the dark. Following secondary antibody incubation, cells were washed twice with 1X PBS and then fixed with ProlongTM Gold antifade reagent with DAPI (P36935; Invitrogen). Stained cells were dried overnight in the dark and imaged using a LSM 700 laser scanning confocal microscope (Carl Zeiss, Toronto, ON, Canada).

Flow Cytometry

BMICs were dissociated into single cells as described and resuspended in phosphate buffered saline (PBS; #311-011-CL; Wisent Inc.) containing 2 mmol/L EDTA (#15575020; Invitrogen). Cell suspensions were then stained with human anti-TRA-1-85-APC (1:50; 130-107-101; Miltenyi Biotec, Auburn, CA, U.S.A.), anti-CD44-PE (1:50; 130-113-342; Miltenyi Biotec), anti-CD133-APC (1:50; 130-112-158; Miltenyi Biotec) or anti-HLA-G-APC (1:100; SAB4700312; Sigma-Aldrich) antibody and incubated for 15 minutes at room temperature. Post-incubation, the viability dye 7-Aminoactinomycin D (7AAD; 1:10; 00-6993-50; Thermo Fisher Scientific) was added to the stained cells to exclude dead cells. Stained cells were then run on a MoFlo XDP Cell Sorter (Beckman Coulter, Mississauga, ON, Canada). Positivity or negativity of staining was determined using analyzed regions from isotype control. For GFP-positive cell sorts, FSC-Height vs. SSC-Height is used as the initial gate to exclude debris. Viability gate is then set using 7-AAD dye to exclude non-

viable cells. Un-transduced control is used to set the gate for expression of GFP, where gate is drawn to distinguish between GFP-negative and GFP-positive populations.

Lentivirus Production

1.5 million HEK293T cells were seeded into 100mm plates and cultured for 24 hours in DMEM high glucose media supplemented with 2mM L-glutamine, 1 mM sodium pyruvate, 1% (5.0 mL) non-essential amino acid solution, 10% (50 mL) FBS, 10 mM (5 mL of 1.0 M) HEPES and 1 mM (550 uL of 1M) sodium butyrate). Next day, culture media was aspirated from HEK293T cells and carefully replaced with 7 mL of fresh supplemented DMEM media. Then, 8 ug of respective transgene-containing (DNA) plasmid (shGFP, shHLA-G1, shHLA-G2, Ctrl-BioID, HLA-G-BioID, AAVS, SPAG9-Knockout (KO)-1, SPAG9-KO2), 4 ug of Gag/pol, and 2 ug of VSVg plasmid was mixed with 500 uL of Opti-MEM in one tube while in a separate tube, 450 uL of Opti-MEM was mixed with 48 ug of Polyethylenimine transfection reagent (PEI) (ratio of 1:3 between amount of PEI: total concentration of DNA). The DNA/Opti-MEM mixture was then added to the PEI/Opti-MEM mixture and incubated for 15 min at room temperature. Post-incubation, the corresponding PEI/DNA mixture solution in 1 mL total volume was then added gently in a dropwise manner to cells, for a final volume of 8 mL per plate (scaled up as needed). Twenty-four hours after adding the transfection mixture to HEK293T cells, the DMEM-supplemented/Opti-MEM/DNA/PEI media was aspirated into a 50 mL Falcon tube and stored at 4°C. DMEM supplemented media was then carefully replaced on HEK293T transfected cells and twenty-four hours later the same process as outlined above was repeated to harvest a second lentiviral batch. Immediately after the third harvest on day 3, the accumulated lentiviral soup was concentrated via ultracentrifugation and resuspended into 1mL of NCC media per viral soup from one plate of HEK293T cells. Lentiviruses were subsequently used for downstream infection or stored long-term at -80°C.

Generation of HLA-G knockdown BMICs

Parental lung (BT478 and BT530) and melanoma (BT673 and BT917) BMICs were infected or transduced with lentivirus containing control (shGFP) or HLA-G specific shRNAs (shHLA-G1 and shHLA-G2) for three

days. Following transduction, cells were selected for 4 days or 2 days using 1ug/mL puromycin for BT478 and BT673 BMICs and 0.5ug/mL puromycin for BT530 and BT917 BMICs. qRT-PCR analysis was used to confirm HLA-G depletion in BMICs. After four days or two days of selection to ensure efficient HLA-G knockdown, cells were dissociated and used for *in vitro* assays or *in vivo* experiments as described below.

Generation of HLA-G overexpressing BMICs

Parental lung (BT478 and BT530) and melanoma (BT673 and BT917) BMICs were infected with lentivirus containing either an empty plasmid encoding for the mutant biotin (Arg118Gly) ligase BirA (BirA*) € or a HLA-G-overexpressing-BirA* plasmid containing the HLA-G coding sequence (CDS) fused in frame with the CDS for BirA* at the amino-terminal end (41-43) for two days. Following infection, cells were selected for 7 days using 1ug/mL puromycin for BT478 and BT673 BMICs and 0.5ug/mL puromycin for BT530 and BT917 BMICs. Western blot, immunocytochemistry and/or flow cytometry analysis was used to confirm HLA-G overexpression in BMICs.

Generation of SPAG9 Knockout BMICs

Control (E) lung (BT478 and BT530) and melanoma (BT673 and BT917) BMICs were infected with lentivirus containing single-gRNA lentiCRISPRv2 constructs targeting AAVS1 while HLA-G OE lung and melanoma BMICs and CRUK0748-XCL cells were infected with lentivirus containing single-gRNA lentiCRISPRv2 constructs targeting AAVS1 or SPAG9 (2 gRNAs), with gRNA sequences obtained from TKOv3 (92). Control and HLA-G OE lung and melanoma BMICs were maintained in puromycin supplemented media as described above while CRUK0748-XCL cells were selected in 2ug/mL puromycin DMEM/F12 supplemented media. Western blot analysis was used to determine efficient SPAG9 KO in cells.

Secondary Sphere Formation Assay

BMICs were dissociated as described above and plated as single cells (200 cells per well) in tumorsphere enriching media and low-binding 96-well plates to prevent cell adhesion. The number of spheres per well were

then manually counted at the four-day incubation time point. Results are illustrated and analyzed for significance using GraphPad Prism 6 software.

Proliferation Assay

BMICs were dissociated as described above and plated as single cells in triplicate at a density of 1000 cells/well in low-binding 96-well plates. Cells were then incubated at 37□C in a humidified atmosphere of 5% CO2. Four days post incubation, PrestoBlue® (#A13261; Invitrogen, Burlington, Canada), a resazurin-based cell viability reagent and fluorescence indicator of cell metabolism, was then added to each well to estimate proliferation four hours prior to measuring fluorescence intensity via FLUOstar Omega Fluorescence 556 Microplate reader (BMG LABTECH, Ortenberg, Baden-Württemberg, Germany) at an excitation and emission wavelength of 540 nm and 590 nm respectively. All results are illustrated and assessed for significance using GraphPad Prism 6 software.

IC_{50} Dose Response Curves

For dose-response assays, drugs were plated using two-fold serial dilutions (20µM - 39 nM) in a 96-well plate, in triplicate at a density of 1000 cells/well, and incubated at 37°C with a humidified atmosphere of 5% CO2 for three days. Vehicle controls for cell death were used in each experiment. Following treatment, 20µL of PrestoBlue® (Invitrogen), was added to each well to estimate proliferation approximately two hours prior to measuring fluorescence intensity via FLUOstar Omega Fluorescence 556 Microplate reader (BMG LABTECH) at an excitation and emission wavelength of 540 nm and 590 nm, respectively. Results were analyzed using Omega analysis software. The half maximal inhibitory concentration (IC_{50}) was determined by plotting percent cell viability by the logarithmic concentration of the drug.

In vitro limiting dilution assay

BMICs were dissociated into single cells as described and seeded in varying cell numbers (1, 5, 15, 25, 50, 75, 100, 150, 200, 275, 375, 400 and 1000 cells/well) in low-binding 96-well plates. Four days post-seeding,

tumorspheres were counted and results plotted using GraphPad Prism 6 software to determine the median stem cell frequencies of breast and melanoma BMICs.

In vivo experiments

All animal experiments were approved and performed according to the guidelines by McMaster University Animal Research Ethics Board (AREB) – AUP Number (19-01-01). NOD SCID Gamma (NSG) mice were used for all experiments. Before marginally invasive surgery, mice were sedated with gas anaesthesia (Isoflurane: 5% induction, 2.5% maintenance). Injections were then performed as previously described for intrathoracic (IT; 500,00 cells) and intracranial (ICr; 200,000 cells) routes (1), with the exception of our experiment involving CRUK0748-XCL AAVS and *SPAG9* KO cells where 1 x 10^6 cells were used for intrathoracic injections. Following euthanization of orthotopically injected mice, each mice group were first perfused to de-vascularize the mice prior to their brains being harvested, processed, minimally (2 weeks) cultured and subjected to flow cytometry analysis to capture and isolate human Tra-1-85 positive or GFP-positive human cell population from bulk mouse brain cells. For intracranial injections, the brains of each respective mice group at mice euthanization were fixed in 10% formalin, paraffin embedded and hemotoxylin & eosin (H&E) stained to histologically analyze tumor areas of each corresponding mice cohort.

For fat pad and subcutaneous injections, single cell suspensions (200,000 and 500,000 cells respectively) were injected into the fourth mammary fat pad or under the skin of the mice right flank respectively (n = 2 each). Mice were observed weekly, and at endpoint, brains as well as orthotopic lung, fat pad and subcutaneous tumors were harvested and either fixed in 10% formalin, paraffin-embedded and subjected to H&E staining or whole brains were harvested and subjected to minimal culturing in tumorsphere media (NCC or SCM or DMEM/F12 supplemented media with 0.2% MycoZapTM Prophylactic) for two weeks to enrich for BMICs prior to flow cytometry analysis. H&E images were obtained using an Aperio Slide Scanner and analyzed using the ImageScope v11.1.2.760 software (Aperio, Rochester, NY, U.S.A.).

For vehicle (DMSO) and DR-1-55 treated HLA-G overexpressing lung and melanoma BMICs intracranial injection experiments, we intracranially injected into NSG brains (n = 5; outliers were exempted from analysis), control (vehicle-treated empty (E) and HLA-G overexpressing (HLA-G OE) lung and melanoma BMICs), and the treatment-refractory cell population of HLA-G overexpressing lung and melanoma BMICs treated *ex vivo* with IC_{80} values of DR-1-55 for three days. When either a control or an experimental animal from both cohorts reached the humane endpoint, a corresponding mouse from the other group was sacrificed simultaneously (matched time endpoints) and their brains were subjected to histological (H&E stain and visualization) analysis.

***In vivo* limiting dilution assay**

BMICs were dissociated into single cells as described and injected intracranially into NSG mice as described above, at dilutions from 500,000 cells per mouse down to 100 cells per mouse (n = 2 mice per BMIC line). Rate of tumor formation was then monitored post-injection. At endpoint, respective mice brains were harvested and fixed with 10% formalin. Fixed tissues were then cut into 5 μM sections on slides and subjected to H&E staining. Stained images were scanned using Aperio Slide Scanner and analyzed using ImageScope v11.1.2.760 software. Brain sections were then assessed for tumor lesions.

***In vivo* imaging**

Bioluminescent imaging of GFP-Luciferase tagged CRUK0748-XCL cells was performed using an In Vivo Imaging System (IVIS) Spectrum (PerkinElmer, MA, U.S.A.) as per the manufacturer's instructions. Imaging and quantification of signals is controlled by the analysis software Living Image (Xenogen). Mice are weighed and injected intraperitoneally with 10μl/g of 15mg/ml solution of D-Luciferin firefly solution (PerkinElmer) in phosphate buffered saline (Invitrogen) 10 minutes before being imaged, and anesthetized (3% induction, 3% maintenance isofluorane). Mice are then placed onto a warmed stage inside the instrument and imaged for a maximum of 2 minutes depending on the tumor size. Regions of interest are quantified as radiance (p/sec/cm2/sr) using Living Image software for a standardized comparison between images.

Ex vivo experiments

BT530 and BT673 single cells were incubated in each of the following conditions: control (E) DMSO-treated, HLAG-OE DMSO-treated and HLAG-OE DR-1-55-treated (at IC80). After a three-day incubation period at 37°C in a humidified atmosphere of 5% CO_2, treatment refractory cell populations were dissociated into single cell suspensions using Liberase (#05401119001; Roche) for cells grown in suspension or TypLE (#12605028; Thermo Fisher) for adherent cells. Mice were sedated with gas anaesthesia (Isoflurane: 5% induction, 2.5% maintenance) and intracranial injections were then performed as previously described (20). Mice were observed weekly, and at matched time endpoint, brains were harvested and fixed in 10% formalin, paraffin-embedded and subjected to hemotoxylin & eosin (H&E) staining. Tumor area measurements were carried out using ImageScope v11.1.2.760 software (Aperio, Rochester, NY, U.S.A.).

Immunohistochemistry

Briefly, tissue slides were deparaffinized by immersion in xylenes 3times for 5 minutes and then rehydrated in decreasing concentrations (100%, 95%, 70%) of alcohol solutions 2 times for 5 minutes each. Antigen retrieval was performed by using an antigen unmasking citrate-based solution (H-3300; Vector Laboratories, Burlington, ON, Canada) at 98°C for 30 minutes in a microwave after which slides were stained with respective primary antibodies (**Dataset S11**) overnight at 4°C. Endogenous peroxidase was blocked using 3% hydrogen peroxide solution (H1009; Sigma-Aldrich, St. Louis, MO, U.S.A.) for 15 minutes at room temperature while endogenous biotin, biotin receptors, and avidin binding sites were blocked using an Avidin/Biotin Blocking Kits (VECTSP-2001; Vector Laboratories) prior to secondary antibody (**Dataset S11**) incubation for 2 hours at room temperature. Slides were washed between each step with either 1X TBS-T and/or 1X TBS. At the end of secondary antibody incubations, slides were stained with DAB Peroxidase (HRP) Substrate Kit (VECTSK4100; Vector Laboratories), counterstained with filtered modified Harris hematoxylin solution (HHS32; Sigma-Aldrich), and differentiated with acid alcohol. Slides were then dehydrated in increasing concentrations (70%, 95%, 100%) of alcohol solutions once for 5 minutes and immersed in xylenes twice for

5 minutes each before being mounted with PolyMount (Polysciences Inc., Warrington, PA, United States) and glass cover slips. Images were captured using Aperio Slide Scanner (Leica Biosystems, Concord, ON, Canada).

LC-MS BioID Analysis

Control and HLA-G overexpressing lung BMICs were treated with biotin for 24 hours to induce biotinylation of proteins proximal to HLA-G. Consistent with published BioID procedures (51), 24 hours of biotin treatment was sufficient to elicit a robust biotinylation signal. Following cell lysis, biotinylated peptides were affinity purified from vehicle-control and biotin-treated HLA-G overexpressing BMICs using streptavidin beads and then samples were resuspended with 20 ul 0.1% formic acid, 1 µl out of 20 µl was injected for LC-MS/MS analysis. Liquid chromatography was conducted using a home-made trap-column (5 cm x 200 µm inner diameter) and a home-made analytical column (50 cm x 75 µm inner diameter) packed with Reprosil-Pur 120 C18-AQ 1.9 µm particles (Dr. Maisch), running a 2hour reversed-phase gradient at 200nl/min on a Thermo Fisher Ultimate 3000 RSLCNano UPLC system coupled to a Thermo QExactive HF quadrupole-Orbitrap mass spectrometer. A parent ion scan was performed using a resolving power of 120,000 and then up to the 30 most intense peaks were selected for MS/MS (minimum ion counts of 1000 for activation), using higher energy collision induced dissociation (HCD) fragmentation. Dynamic exclusion was activated such that MS/MS of the same m/z (within a range of 10ppm; exclusion list size=500) detected twice within 5s were excluded from analysis for 30s. LC-MS data were searched against a UniProt human protein database (Ver 2017-06, 42,173 entries) for protein identification and quantification by Protein Discover software (Thermo), with peptide FDR < 0.01 and protein FDR <0.01 as cut-off thresholds. Resulting proteins from BioID samples and controls were further analyzed using SAINT or significance analysis of interactome (52) to identify significant interactors for the bait protein based on BFDR scores.

KMplotter Database Analyses

The KMplotter database was used to analyze the expression of HLA-G and SPAG9 independently or together (https://kmplot.com/analysis/index.php?p=service&cancer=pancancer_rnaseq and

https://kmplot.com/analysis/index.php?p=service&cancer=lung). Kaplan-Meier survival analysis using "auto best cut-off" on the portal was conducted by differentiating between samples with high versus low HLA-G, SPAG9 and HLA-G with SPAG9 expression. Analyzed results obtained was downloaded and included in the manuscript.

Statistical Analyses

All experiments were performed in duplicates or triplicates. Applicable data were analyzed and represented using GraphPad Prism 6 software. Data are presented as means±S.E.M. Statistical tests were also completed using GraphPad Prism 6 software. Unpaired Student's t-tests were used for statistical analysis of two data sets and one-way analysis of variance with Tukey/Newman–Keuls test was used for statistical analysis of more than two data sets, with a p-value < 0.05 deemed as statistically significant. Grubbs' test was used to calculate for outliers, which were excluded from analysis.

References

1. Singh M, Venugopal C, Tokar T, McFarlane N, Subapanditha MK, Qazi M, et al. Therapeutic Targeting of the Premetastatic Stage in Human Lung-to-Brain Metastasis. Cancer Res. 2018;78(17):5124-34. Epub 2018/07/09. doi: 10.1158/0008-5472.CAN-18-1022. PubMed PMID: 29986997.
2. Bustin SA, Benes V, Garson JA, Hellemans J, Huggett J, Kubista M, et al. The MIQE guidelines: minimum information for publication of quantitative real-time PCR experiments. Clin Chem. 2009;55(4):611-22: Epub 20090226. doi: 10.1373/clinchem.2008.112797. PubMed PMID: 19246619.

A

B

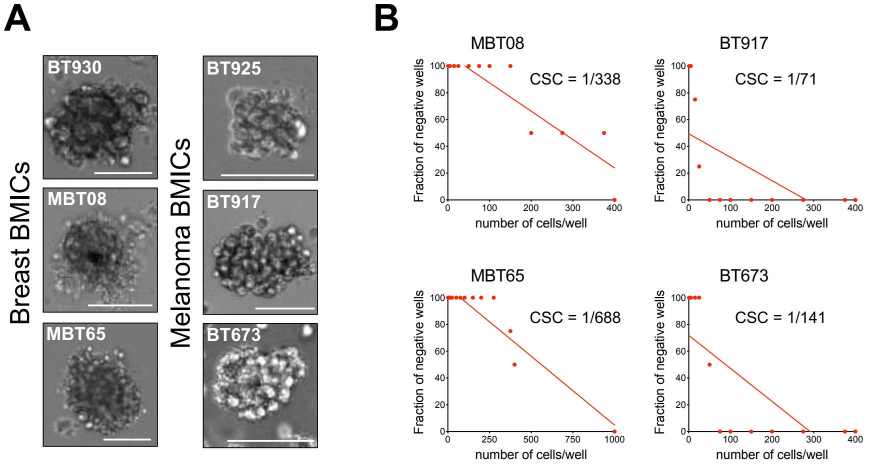

C

In vitro LDA	
Breast (Br) BMICs	Stem cell frequency
MBT08	1 in 338
MBT65	1 in 688
Melanoma (Mel) BMICs	Stem cell frequency
BT917	1 in 71
BT673	1 in 141

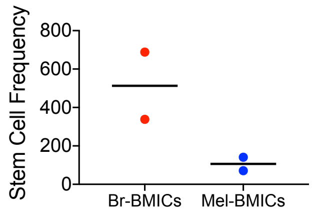

D

Sample Identifier	CD44%	CD133%
BT923	99.98	0.19
BT930	97.25	1.82
MBT08	71.83	0.22
MBT65	84.40	0.09
BT925	98.80	0.87
BT917	67.42	0.05
BT673	84.99	0.73

E

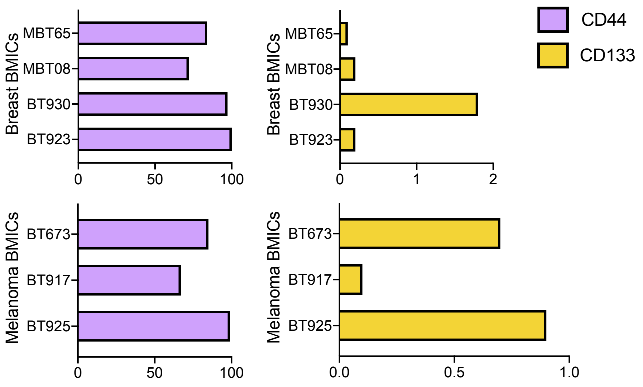

Supplementary Figure 1: *In vitro* characterization of patient-derived breast and melanoma BMICs: A. Representative bright field images of BMIC spheres enriched from breast- and melanoma-brain metastatic tumors. **B, C.** *In vitro* limiting dilution analysis (LDA) to determine the stem cell frequencies of breast and melanoma BMICs. **D, E.** Surface expression of cancer stem cell markers (CD44 and CD133) in breast and melanoma BMICs. Scale bar = 100um.

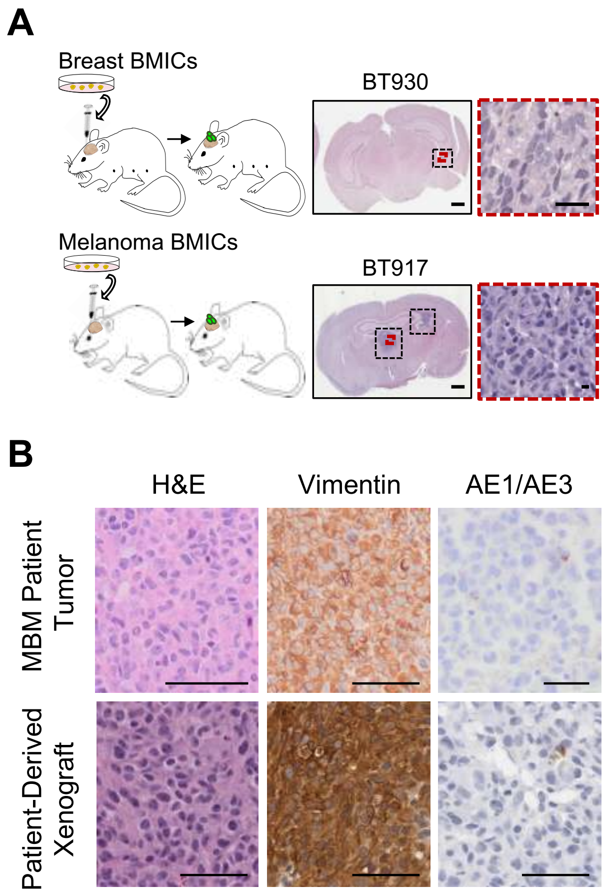

Supplementary Figure 2: *In vivo* characterization of patient-derived breast and melanoma BMICs: A. Right – schematic illustration of the tumor engraftment process (intracranial injections) of breast and melanoma BMICs; Left – Representative hematoxylin and eosin (H&E) images of breast (BT930) and melanoma (BT917) reformed brain tumors. Number of BMICs = 500,000 each. Scale bar = 1000um. 20X Scale bar = 10um. **B.** H&E-stained and molecular markers (Vimentin and AE1/AE3) stained images of melanoma (BT917) patient-derived xenograft in comparison to original melanoma-BM patient tumor. 20X Scale bar = 20um.

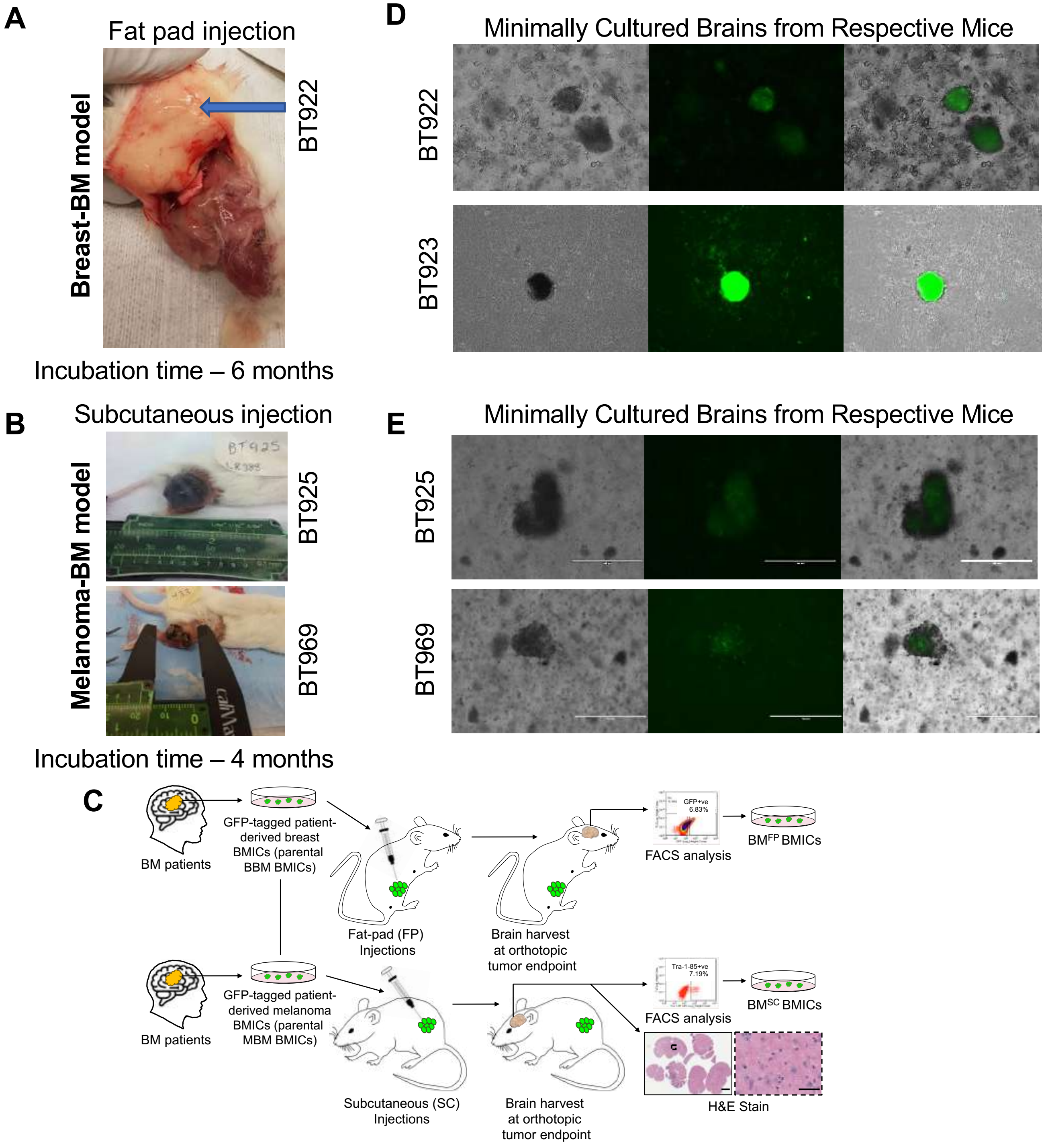

Supplementary Figure 3: Capture of breast and melanoma BMICs at the pre-metastatic stage of BM development. Representative image shown of **A.** breast BMICs (BT922- 200,000 cells) injected into the fat pad of mice (n = 2) and **B.** melanoma BMICs (BT925 and BT969 - 500,000 cells each) subcutaneously injected into mice (n = 4). **C.** Schematic illustration of the process performed to capture pre-metastatic breast (BM^{FP}) and melanoma (BM^{SC}) BMICs. Brain metastatic tumors from breast and melanoma patients were surgically removed and enriched for BMICs, which were then GFP-tagged and injected into respective orthotopic sites (fat pad and right flank respectively) of mice. At orthotopic tumor endpoints, brains of respective mice were harvested and either fixed and H&E stained and/or processed, minimally cultured and sorted via flow cytometry for GFP-positive BM^{FP} and BM^{SC} BMICs. 0.4X Scale bar = 2000um. 20X Scale bar = 100um. **D, E.** Cultured GFP-positive BM^{FP} and BM^{SC} BMICs retain their secondary sphere-forming capacities. Scale bar = 400um.

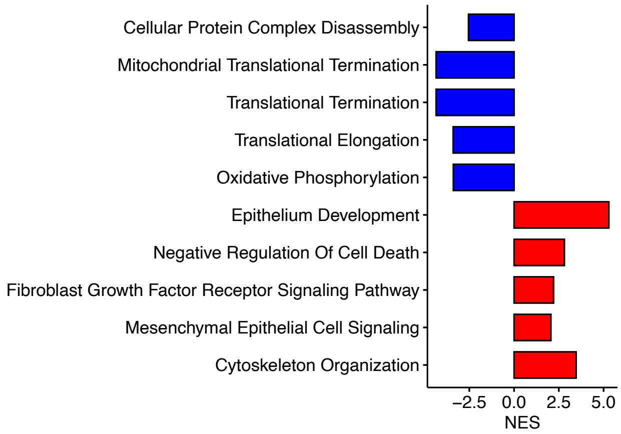

Supplementary Figure 4: Biological processes associated with pre-metastatic breast and melanoma BMICs. Bar plots showing significantly up- and down-regulated biological processes associated with the differentially expressed genes (DEGs) between pre-metastatic and non-pre-metastatic breast and melanoma BMICs.

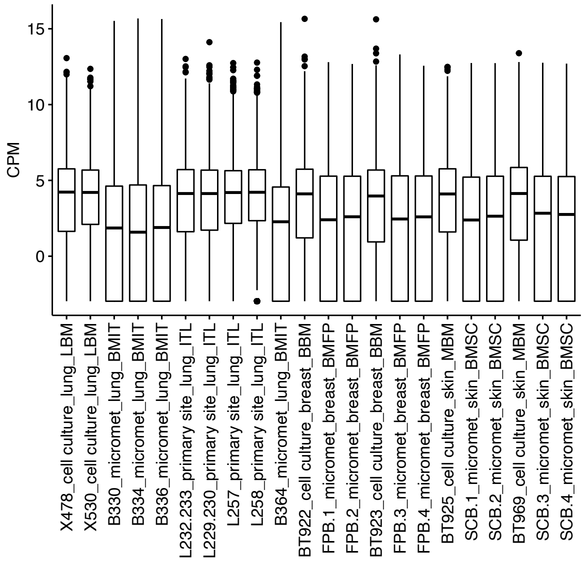

Supplementary Figure 5: Normalized data of pre-metastatic and parental lung, breast and melanoma BMICs. Bar plots showing the normalized data of pre-metastatic (BM^{IT}, BM^{FP}, BM^{SC}) and non-pre-metastatic (LBM, BBM, MBM) lung, breast and melanoma BMICs.

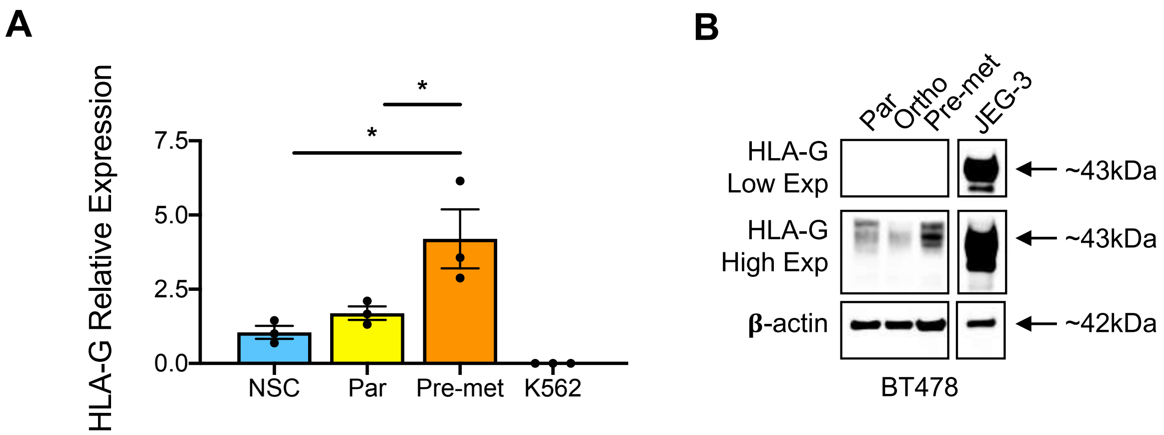

Supplementary Figure 6: qRT-PCR and western blot analysis of HLA-G expression in parental and pre-metastatic lung BMICs. A. qRT–PCR analysis of HLA-G expression in neural stem cells (NSC), parental (Par) and pre-metastatic (Pre-met) lung (BT478) BMICs and the immortalized myelogenous leukemia line K562 used as a negative control. **B.** Western blot analysis of HLA-G expression in Par, orthotopic (Ortho –intrathoracic lung tumors) and pre-met BMICs with JEG-3 (choriocarcinoma) cells used as a positive control. **β**-actin serves as a loading control. p value * 0.0117 between NSC and pre-met and * 0.04 between par and pre-met.

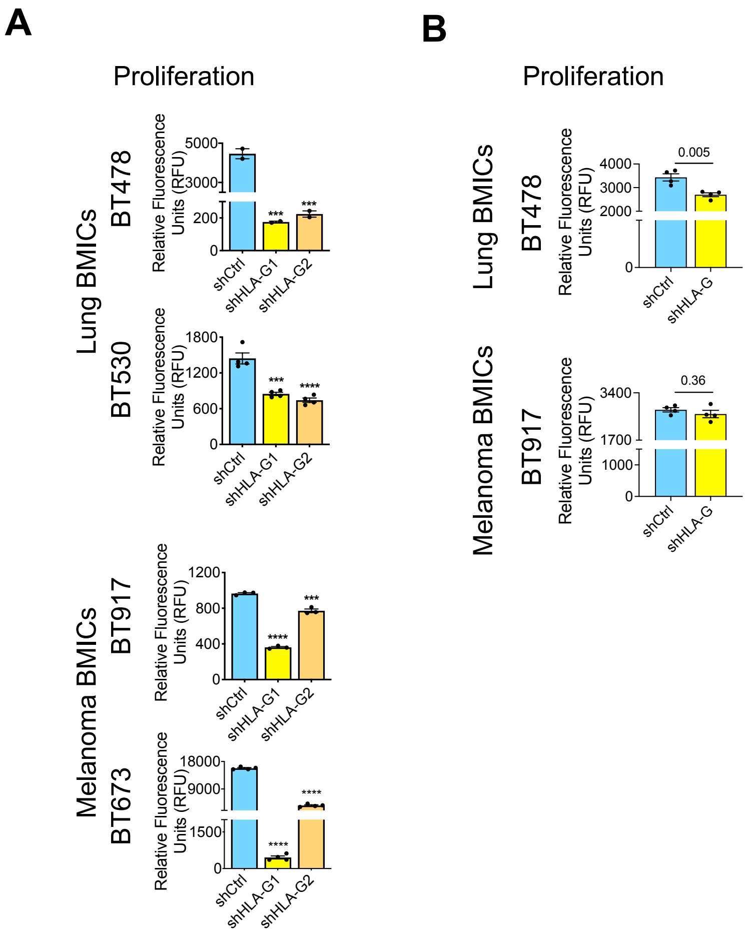

Supplementary Figure 7: HLA-G depletion reduces the proliferation of lung and melanoma BMICs. A. *In vitro* characterization (proliferation assays) of control (shCtrl) and HLA-G knockdown (shHLA-G1 and shHLA-G2) lung (BT478; BT530) and melanoma (BT917; BT673) BMICs. p values for BT478 lung BMICs proliferation assessed as relative fluorescence units (RFU) *** 0.0004 and 0.0004; p values for BT530 lung BMICs RFU *** 0.0001 and **** <0.0001; p values for BT917 melanoma BMICs RFU **** <0.0001 and *** 0.0001; p values for BT673 melanoma BMICs **** <0.0001 and **** <0.0001. **B.** Bar plot showing proliferation of BT478- shCtrl and shHLA-G cells and proliferation of BT917- shCtrl and shHLA-G cells used for *in vivo* experiments two days post selection. p values as shown.

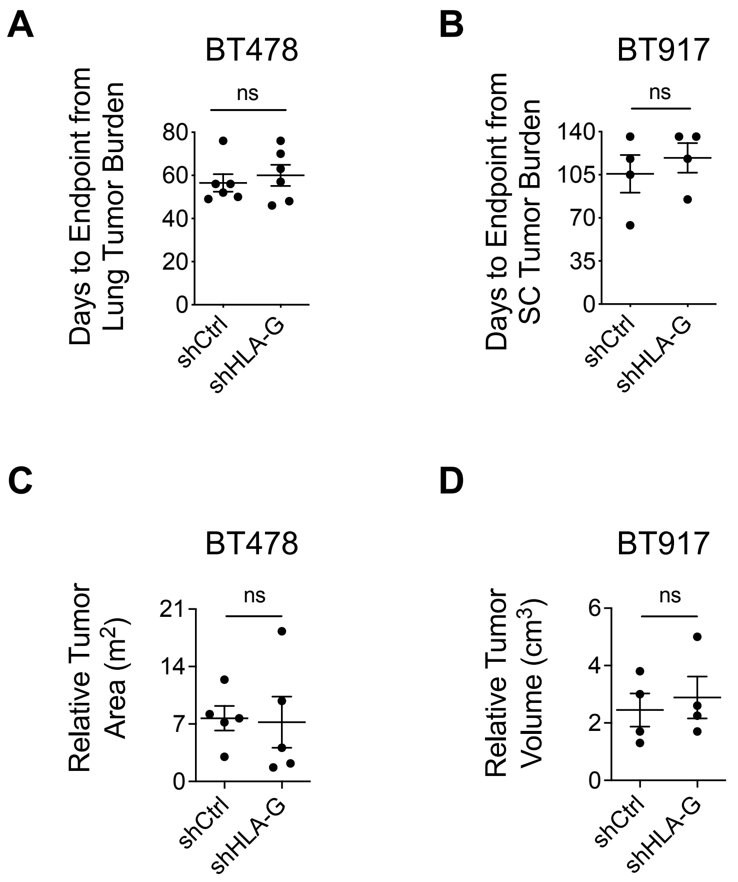

Supplementary Figure 8: HLA-G knockdown does not affect orthotopic lung and melanoma tumor formation. A. Scatter-dot plot showing days to orthotopic tumor endpoints from lung tumor burden of BT478- shCtrl and shHLA-G mice (n = 6 each). p value ns = 0.59. **B.** Scatter-dot plot showing days to orthotopic tumor endpoints from melanoma (flank) tumor burden of BT917- shCtrl and shHLA-G mice (n = 4 each). p value ns = 0.39. **C.** Scatter-dot plot showing relative orthotopic tumor area from lung tumor burden of BT478- shCtrl and shHLA-G mice (n = 5 each). p value ns = 0.89. **D.** Scatter-dot plot showing relative orthotopic tumor volume from melanoma (flank) tumor burden of BT917- shCtrl and shHLA-G mice (n = 4 each). p value ns = 0.66.

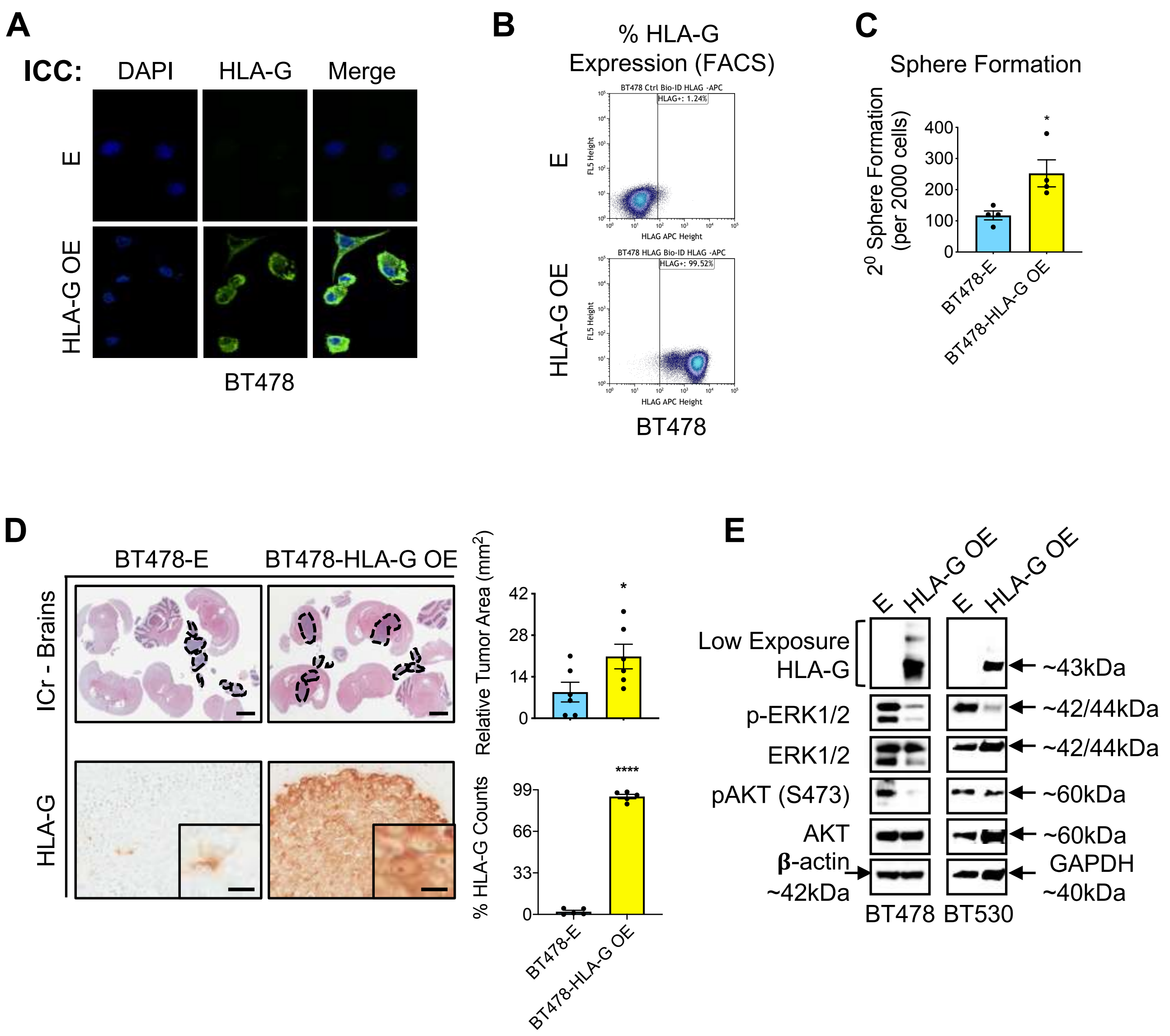

Supplementary Figure 9: HLA-G overexpression effects on ERK and AKT Signaling Pathways. A. Immunocytochemistry (ICC) of lung (BT478)-control (E) and HLA-G overexpressing (OE) BMICs stained with HLA-G antibody and DAPI. **B.** Flow cytometry analysis to determine membrane HLA-G expression in BT478-E and HLA-G OE BMICs. **C.** *In vitro* functional characterization (secondary (2^0) sphere formation assays) of control (BT478-E) and HLA-G overexpressing (BT478 HLA-G OE) lung BMICs. p value * = 0.03. **D.** H&E and HLA-G stained images of brain tissues of mice intracranially (ICr) injected with BT478 Ctrl and HLA-G OE lung BMICs. Scale bar = 2000um. p value relative tumor area * = 0.048; % HLA-G counts **** <0.0001. To the right are bar graphs depicting relative tumor area (mm^2) and % respective stained counts for BT478 Ctrl and HLA-G OE lung tumors. **E.** Western blot analysis of BT478 and BT530 E and HLA-G OE BMICs for HLA-G, p-ERK1/2, ERK1/2, pAKT (S473) and AKT with **β**-actin and GAPDH serving as controls.. All experiments were conducted in duplicate or triplicate.

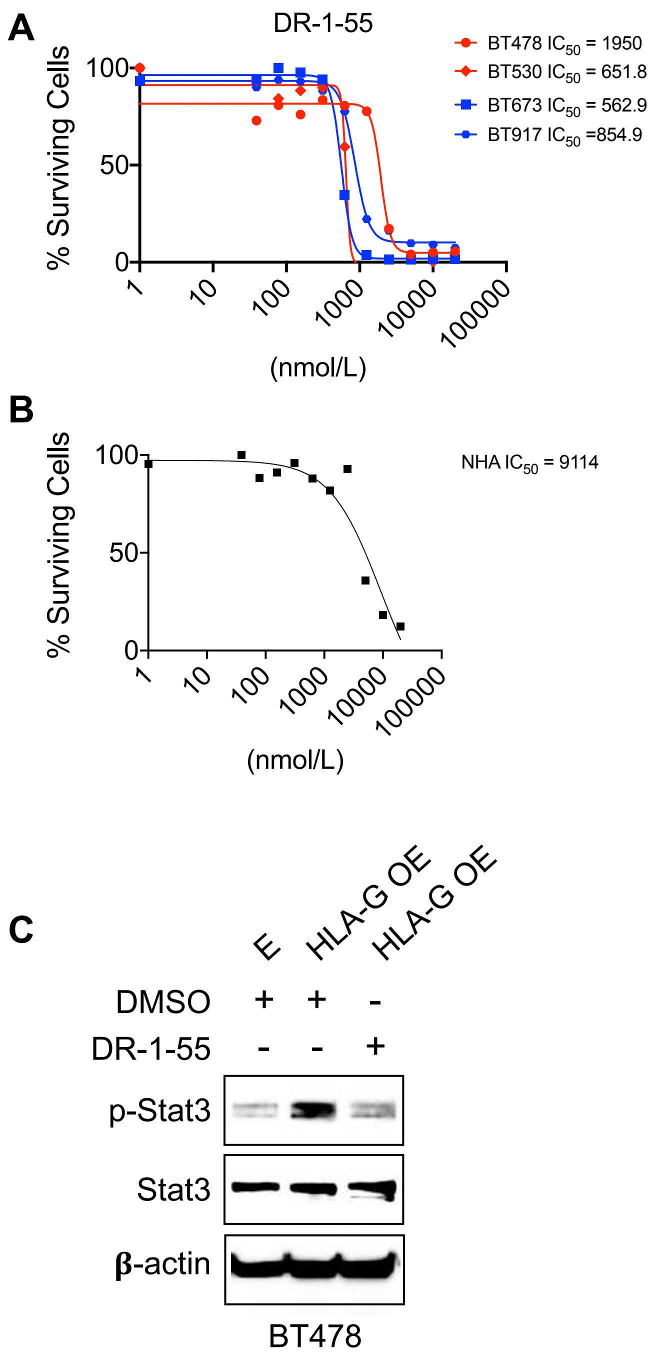

Supplementary Figure 10: DR-1-55 dose-response curves and effect on STAT3 signaling in BMICs. A. *In vitro* IC_{50} dose response curves of lung (BT478 and BT530) and melanoma (BT673 and BT917) BMICs treated with the STAT3 inhibitory drug (DR-1-55) in two-fold serial dilutions (20μM - 39 nM) in 96-well plates, in triplicate at a density of 1000 cells/well. **B.** . *In vitro* IC_{50} dose response curves of normal human astrocytes (NHA) treated with DR-1-55 in two-fold serial dilutions (20μM - 39 nM) in 96-well plates, in triplicate at a density of 1000 cells/well. **C.** Western blot analysis of p-STAT3 (Y705) and STAT3 expression in BT478 control (E) and HLA-G OE BMICs treated with IC_{80} concentrations of DR-1-55 with **β**-actin serving as loading control.

GSE110495 Lung BMIC Dataset		
**Gene**	**Mean normalized read count**	
	Parental lung BMICs	Pre-metastatic lung BMICs
LILRB1	0	0
LILRB2	0	0
HLA-G	0.53	6.36

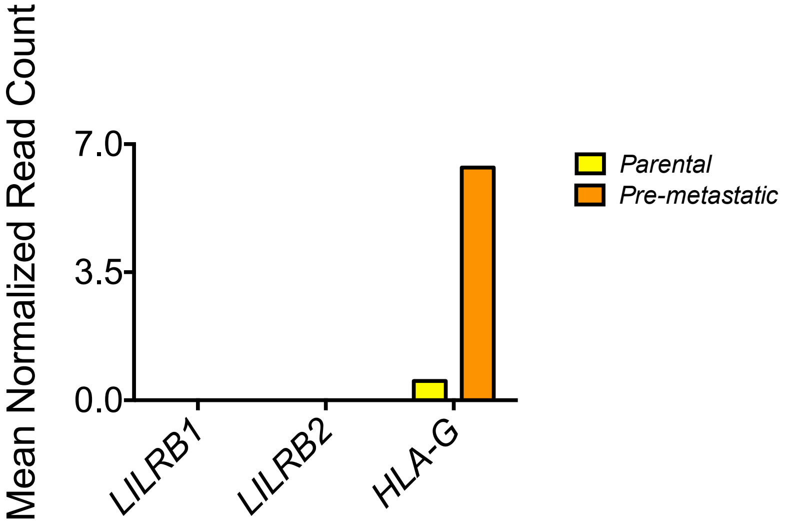

Supplementary Figure 11: Mean normalized read count of *LILRB1*, *LILRB2* and *HLA-G in* parental and pre-metastatic lung BMICs. Mean normalized read count = 0) or pre-metastatic (mean normalized read count = 0) lung BMICs when compared to the expression values for HLA-G in parental (mean normalized read count = 0.53) or pre-metastatic (mean normalized read count = 6.36) lung BMICs.

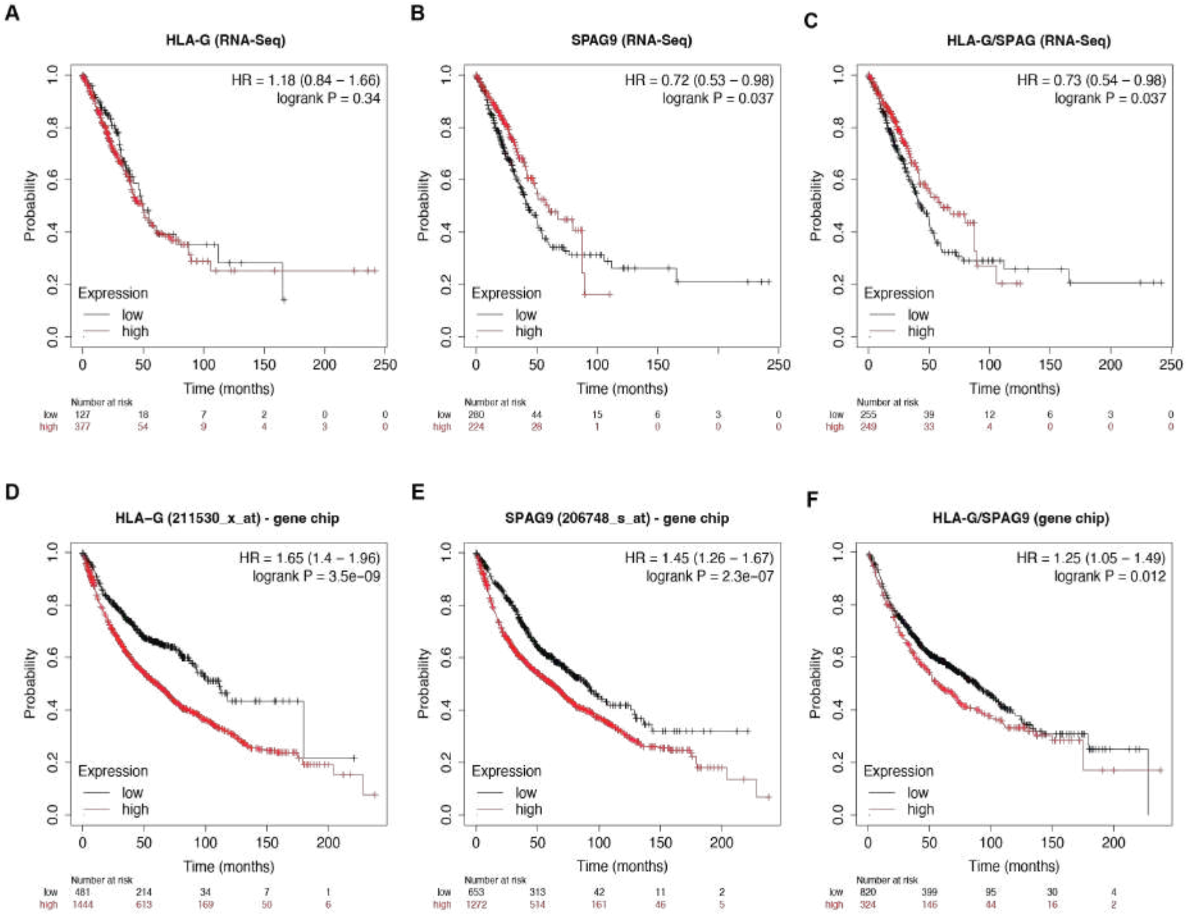

Supplementary Figure 12: High HLA-G and SPAG9 Expression Independently or Together Correlates with Poor Survival Outcomes in Lung Cancer Patients. The effects of high **A.** HLA-G, **B.** SPAG9 or **C.** high HLA-G and SPAG9 expression on poor overall survival in lung cancer patients. KMplotter RNA-seq dataset was used for these analyses. Kaplan–Meier survival curves also showing the effects of high **D.** HLA-G, **E.** SPAG9 or **F.** high HLA-G and SPAG9 expression on poor overall survival using a different dataset of lung cancer patients (KMplotter gene chip dataset was used for these analyses). Statistical significance was determined by log-rank test and p-values are indicated.

Table S1: Breast and melanoma patients information

Sample Identifier	Gender	Age	Tumor Removal Area	Primary Tumor	BMICs used for RNA sequencing	BMICs established into primary cell lines
BT922	F	51	Right Parietal/Temporal	Breast	Yes	No
BT923	F	43	Left Parietal	Breast	Yes	No
BT930	F	55	Right Parietal	Breast	No	No
MBT08	F	76	Left Frontal	Breast	No	No
MBT65	F	59	Right Parieto-Occipital	Breast	No	No
BT925	F	75	Right Occipital	Melanoma	Yes	No
BT969	F	58	Right Frontal Cerebral Lobe	Melanoma	Yes	No
BT917	M	76	Left Parietal	Amelanotic Melanoma	No	Yes
BT673	M	58	Right Frontal	Melanoma	No	Yes

Table S2: *In vivo* intracranial limiting dilution assays of melanoma BMICs; Related to Figure S2		
BT917		
**Cell #**	**# of mice injected**	**Tumor Frequency**
100	2	2
1,000	2	2
10,000	2	2
100,000	2	2
500,000	2	2

Table S3: List of gene-specific primers used in study

Primers	5' - 3' Sequence	Annealing Temperature (ºC)
HLA-G Fwd	CCACCACCCTGTCTTTGACT	62
HLA-G Rev	TGGCACGTGTATCTCTGCTC	
SPAG9 Fwd	GGCTGTAGAACAGGAGGATGAG	60
SPAG9 Rev	TGGCGCATCTGTAACCTTCA	
CRK Fwd	GCTCTGATTGGAGGTCGGTG	60
CRK Rev	TGTTGATCCAGCAGACGGAC	
